# XPF-like domain in human SHOC1 is required for crossover formation and protecting autosome from MSUC

**DOI:** 10.1093/nar/gkag558

**Published:** 2026-06-08

**Authors:** Yuxiang Zhang, Qian Sun, Zhiyong Ji, Ren Mo, Yuchuan Zhou, Jingpeng Zhao, Shuai Xu, Na Li, Yifan Sun, Haowei Bai, Erlei Zhi, Sha Han, Huixing Chen, Jing Zhang, Dewei Qian, Xinjie Bu, Yuhua Huang, Ruhui Tian, Ying Guo, Jinxing Lv, Liangyu Zhao, Chao Yang, Fujun Zhao, Peng Li, Zhi Zhou, Zheng Li, Chencheng Yao

**Affiliations:** Department of Andrology, Center for Men’s Health, Department of ART, Institute of Urology, Urologic Medical Center, Shanghai Key Laboratory of Reproductive Medicine, Shanghai General Hospital, Shanghai Jiao Tong University School of Medicine, Shanghai 200080, China; Department of Developmental and Regenerative Biology, School of Life Science and Technology, ShanghaiTech University, Shanghai 200120, China; State Key Laboratory of Reproductive Medicine and Offspring Health, Nanjing Medical University, Nanjing 211166, China; Department of Reproductive Medicine, The First Affiliated Hospital of Xiamen University, School of Medicine, Xiamen University, Xiamen 361003, China; Department of Urology, Inner Mongolia People’s Hospital, Inner Mongolia Urological Institute, Hohhot, Inner Mongolia 010017, China; The International Peace Maternity and Child Health Hospital, Shanghai Key Laboratory of Embryo Original Disease, School of Medicine, Shanghai Jiao Tong University, Shanghai 200030, China; Department of Andrology, Center for Men’s Health, Department of ART, Institute of Urology, Urologic Medical Center, Shanghai Key Laboratory of Reproductive Medicine, Shanghai General Hospital, Shanghai Jiao Tong University School of Medicine, Shanghai 200080, China; Department of Andrology, Center for Men’s Health, Department of ART, Institute of Urology, Urologic Medical Center, Shanghai Key Laboratory of Reproductive Medicine, Shanghai General Hospital, Shanghai Jiao Tong University School of Medicine, Shanghai 200080, China; Department of Andrology, Center for Men’s Health, Department of ART, Institute of Urology, Urologic Medical Center, Shanghai Key Laboratory of Reproductive Medicine, Shanghai General Hospital, Shanghai Jiao Tong University School of Medicine, Shanghai 200080, China; Department of Andrology, Center for Men’s Health, Department of ART, Institute of Urology, Urologic Medical Center, Shanghai Key Laboratory of Reproductive Medicine, Shanghai General Hospital, Shanghai Jiao Tong University School of Medicine, Shanghai 200080, China; Department of Andrology, Center for Men’s Health, Department of ART, Institute of Urology, Urologic Medical Center, Shanghai Key Laboratory of Reproductive Medicine, Shanghai General Hospital, Shanghai Jiao Tong University School of Medicine, Shanghai 200080, China; Department of Andrology, Center for Men’s Health, Department of ART, Institute of Urology, Urologic Medical Center, Shanghai Key Laboratory of Reproductive Medicine, Shanghai General Hospital, Shanghai Jiao Tong University School of Medicine, Shanghai 200080, China; Department of Andrology, Center for Men’s Health, Department of ART, Institute of Urology, Urologic Medical Center, Shanghai Key Laboratory of Reproductive Medicine, Shanghai General Hospital, Shanghai Jiao Tong University School of Medicine, Shanghai 200080, China; Department of Andrology, Center for Men’s Health, Department of ART, Institute of Urology, Urologic Medical Center, Shanghai Key Laboratory of Reproductive Medicine, Shanghai General Hospital, Shanghai Jiao Tong University School of Medicine, Shanghai 200080, China; Reproductive Medicine Center, The Sixth Affiliated Hospital, Sun Yat-sen University, 26 Yuancun-er-heng Road, Guangzhou 510655, China; Department of Urology, The Affiliated Taizhou Peoples Hospital of Nanjing Medical University, Taizhou School of Clinical Medicine, Nanjing Medical University, Taizhou 225300, China; Department of Urology, The Affiliated Taizhou Peoples Hospital of Nanjing Medical University, Taizhou School of Clinical Medicine, Nanjing Medical University, Taizhou 225300, China; Department of Andrology, Center for Men’s Health, Department of ART, Institute of Urology, Urologic Medical Center, Shanghai Key Laboratory of Reproductive Medicine, Shanghai General Hospital, Shanghai Jiao Tong University School of Medicine, Shanghai 200080, China; Department of Andrology, Center for Men’s Health, Department of ART, Institute of Urology, Urologic Medical Center, Shanghai Key Laboratory of Reproductive Medicine, Shanghai General Hospital, Shanghai Jiao Tong University School of Medicine, Shanghai 200080, China; The International Peace Maternity and Child Health Hospital, Shanghai Key Laboratory of Embryo Original Disease, School of Medicine, Shanghai Jiao Tong University, Shanghai 200030, China; Department of Reproduction, The Fourth Affiliated Hospital of Soochow University (Suzhou Dushu Lake Hospital), Suzhou 215000, China; Department of Urology, The Fifth Affiliated Hospital of Sun Yat-sen University, Zhuhai 519000, China; Department of Urology, First Affiliated Hospital of Nanjing Medical University, Nanjing 210029, China; Department of Andrology, Center for Men’s Health, Department of ART, Institute of Urology, Urologic Medical Center, Shanghai Key Laboratory of Reproductive Medicine, Shanghai General Hospital, Shanghai Jiao Tong University School of Medicine, Shanghai 200080, China; Department of Andrology, Center for Men’s Health, Department of ART, Institute of Urology, Urologic Medical Center, Shanghai Key Laboratory of Reproductive Medicine, Shanghai General Hospital, Shanghai Jiao Tong University School of Medicine, Shanghai 200080, China; Department of Developmental and Regenerative Biology, School of Life Science and Technology, ShanghaiTech University, Shanghai 200120, China; Department of Andrology, Center for Men’s Health, Department of ART, Institute of Urology, Urologic Medical Center, Shanghai Key Laboratory of Reproductive Medicine, Shanghai General Hospital, Shanghai Jiao Tong University School of Medicine, Shanghai 200080, China; State Key Laboratory of Reproductive Medicine and Offspring Health, Nanjing Medical University, Nanjing 211166, China; Department of Urology, The Affiliated Taizhou Peoples Hospital of Nanjing Medical University, Taizhou School of Clinical Medicine, Nanjing Medical University, Taizhou 225300, China; Department of Andrology, Center for Men’s Health, Department of ART, Institute of Urology, Urologic Medical Center, Shanghai Key Laboratory of Reproductive Medicine, Shanghai General Hospital, Shanghai Jiao Tong University School of Medicine, Shanghai 200080, China

## Abstract

During meiosis, ZMM proteins play essential roles in stabilizing the recombination intermediates and promoting crossover (CO) formation. In mice, shortage in chiasmata 1 (SHOC1) forms a trimeric complex with the other two ZMM proteins, SPO16 and TEX11, to bind recombination intermediates after strand invasion. Although genetic variants of SHOC1 are clinically associated with male infertility, their conserved functions in human gametogenesis remain enigmatic. Here, we delineated species-specific divergences between human and mouse SHOC1 complex and identified a missense variant within the XPF-like domain in SHOC1 (p.Q590R). This variant impaired DNA double-strand breaks repair by compromising its ability to bind branched DNA structures and the recruitment of crucial proteins to recombination intermediates, ultimately abolishing CO formation. Furthermore, the variant disrupted dynamic chromatin structure in pachytene spermatocytes and induced synapsis defects. Importantly, the XPF-like domain in SHOC1 was revealed to prevent autosome intrusion into the sex body compartment, thereby protecting critical autosomal loci from meiotic silencing of unsynapsed chromatin (MSUC). Overall, our study underscores the critical role of the XPF-like domain in human SHOC1 in CO formation and in protecting autosomes from MSUC.

## Graphical Abstract

**Graphical Abstract:**
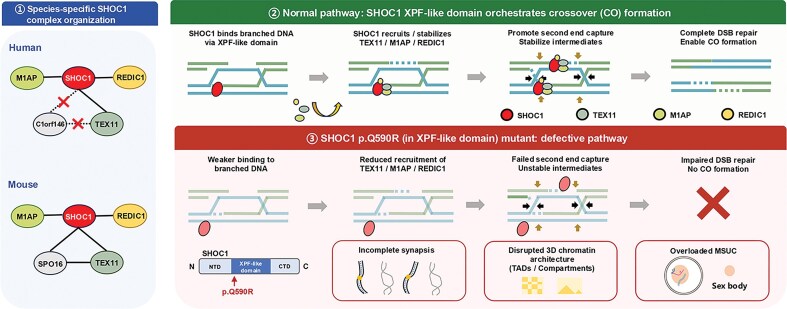

## Introduction

Infertility affects an estimated 15% of couples of reproductive age globally, presenting a significant medical and societal challenge [1]. Male infertility accounts for ~50% of these cases, with non-obstructive azoospermia (NOA) being the most severe type [2]. Meiotic arrest, a subtype of NOA, is widely known to have multiple genetic origins, including chromosome abnormalities, Y chromosome microdeletions, and monogenic variants [3, 4]. However, the pathogenic mechanisms of genetic disorder-induced meiotic arrest remain elusive.

Meiosis is essential for the production of haploid gametes in mammals, during which homologs undergo pairing, synapsis, and recombination. Meiotic recombination is orchestrated through a sophisticated sequence of events. Programmed DNA double-strand breaks (DSBs) are formed by the SPO11 enzyme during the early stages of meiotic prophase I and are quickly processed to generate 3′ single-stranded DNA (3′-ssDNA) overhangs [5, 6]. These overhangs are subsequently enveloped by the single-strand DNA binding protein complex, replication protein A (RPA), and the RAD51/DMC1 recombinase complex. The RPA complex collaborates with meiosis-specific proteins such as MEIOB and SPATA22 to facilitate the recombination process [5–7]. Nascent displacement loops (D-loops) migrate toward the direction of repair synthesis. One resected DSB end can be cross-linked with a partner duplex in a stable and discrete state termed single-end invasion (SEI). In yeast, the SEI intermediates rely on the ZMM factors, including Zip1, Zip2, Zip3, Zip4, Msh4–Msh5 (the MutSγ complex), Mer3, and Spo16, to stabilize nascent joint molecules and coordinately promote polymerization of synaptonemal complex (SC) [8, 9]. These recombination intermediates are bound by RNF212 and HEI10 (an ortholog of Zip3) to regulate their stability and further recruit CDK2 and MLH1–MLH3, and are ultimately repaired into class Ⅰ crossovers (COs) [10, 11].

Three ZMM proteins, Zip2, Zip4, and Spo16, form a functionally conserved protein complex known as “ZZS,” which was initially described in yeast [12]. In mice, all three ZZS proteins localize to chromosome axes as discrete foci and have similar foci kinetics during meiotic prophase I [13]. Notably, mouse shortage in chiasmata 1 (SHOC1), an ortholog of yeast Zip2, has been reported to play indispensable roles in spermatogenesis [14–16]. During meiosis, SHOC1 is recruited to D-loops following strand invasion, where it binds specific DNA to catalytically stabilize recombination intermediates and drive DNA synthesis. Additionally, SHOC1 recruits SPO16 to maintain the stabilization of SHOC1 and proper localization of TEX11 (ortholog of Zip4) and MSH4 [13, 14, 17]. MSH4 and MSH5 form a clamp-like heterodimer to stabilize the DNA structure associated with strand invasion. Moreover, they are responsible for the recruitment of MLH1 and MLH3 to the CO sites in pachytene [18, 19]. *Shoc1*^−/−^ and *Spo16*^−/−^ mice exhibited severe defects in synapsis and meiotic arrest at zygotene-like or early pachytene-like stage without CO formation [13, 14], whereas knockout of *Tex11* in mice caused a milder defect in synapsis and reduced CO formation, leading to meiotic metaphase I (MMI) arrest [20, 21]. Recently, two novel proteins M1AP and REDIC1, have been shown to have similar locations and functions to the ZZS proteins in facilitating CO formation and meiotic progression in males [22, 23].

The XPF-like domain, which is indispensable for binding with DNA structures, is conserved in yeast Zip2, mouse SHOC1 (mSHOC1), as well as human C9orf84 (also referred to SHOC1). The XPF-like proteins, including MUS81, EME1, FANCM, and SHOC1, are distinguished by a helicase domain, an ERCC4-like nuclease domain, and a conserved Ercc4-helix-hairpin-helix (HhH)_2_ core [15]. These proteins are proven to be fundamental in meiotic recombination and typically function as heterodimers, including XPF–ERCC1 complex, MUS81–EME1/2 complex, FANCM–FAAP24 complex, and SHOC1–SPO16 complex [13]. Specifically, Mus81–Eme1 complexes are revealed to be required for resolution of double Holliday junctions into class II COs [24]. Additionally, FANCM–FAAP24 complexes are shown to promote class I interfering COs and suppress class II non-interfering COs [25]. In mice, SHOC1–SPO16 complex cooperates with MSH4–MSH5 to recognize and stabilize early recombination intermediates, recruit downstream CO factors to promote the formation of class Ⅰ COs, and coordinate the formation of COs with assembly of the SC [13]. It has been demonstrated that recombinant XPF-like domain in human SHOC1 preferentially binds branched DNA but lacks detectable endonuclease activity *in vitro* [17]. However, the *in vivo* roles and critical residues of the XPF-like domain in SHOC1 during meiosis still remain unclear.

Herein, we investigated the differences between human and mSHOC1 complexes and identified a missense variant within the XPF-like domain in *SHOC1* (c.A1769G:p.Q590R) that was associated with NOA. Intriguingly, the knock-in (KI) mice mimicking the patient’s variants showed defects in DSBs recombination and homologous chromosome synapsis. Furthermore, abnormal silencing of unsynapsed autosomes in sex body occurred in the pachytene spermatocytes of *Shoc1* KI mice. Overall, our study offers novel insights into meiotic recombination and provides a prospective molecular target for the clinical diagnosis and treatment of infertility.

## Materials and methods

### Study participants

The experiments performed in human were approved by the ethics committee of Shanghai General Hospital (2022SQ294). Written informed consent was obtained from the donors for the use of their clinical data and testicular tissues for research purposes. For the initial cohort, a total of 1072 idiopathic NOA patients were enrolled from the Department of Andrology, Urologic Medical Center, Shanghai General Hospital, Shanghai Jiao Tong University School of Medicine, Shanghai, China. Physical examination and repeated semen analyses following centrifugation were conducted and assessed in accordance with the guidelines of the World Health Organization (fifth edition) [26]. Known causal factors for male infertility, including orchitis, cryptorchidism, varicocele, radiation, chemotherapy, and testicular cancer, were excluded. Genetic screening, including karyotype and Y chromosome microdeletions, was performed in the patients with NOA. The definition of the testicular phenotype was based on multiple testis biopsies (one fragment was analyzed by the pathologist who described the histological picture, and the remaining fragments were analyzed by an embryologist who searched for spermatozoa). Ultimately, 171 patients with meiotic arrest were subjected to whole-exome sequencing (WES) in this study. Additionally, patients with obstructive azoospermia (OA), characterized by normal spermatogenesis but seminal tract obstruction, were included as positive controls.

### WES and Sanger sequencing

Genomic DNA was isolated from peripheral blood samples using a TIANamp Blood DNA Kit (TIANGEN) according to the manufacturer’s protocol. Genomic DNA was subjected to WES performed by the company (Shanghai Yuyin Biotechnology Co., Ltd) on the HiSeq2000 sequencing platform (Illumina Inc., San Diego, CA, USA), as described previously [27, 28]. WES data analysis was performed using the Genome Analysis Toolkit. Briefly, after adaptors were removed, the WES raw reads after removing adaptors were aligned to the human genome (GRCh37/hg19) using the Burrows–Wheeler Aligner [29], followed by removal of the polymerase chain reaction (PCR) duplicates and sorting using Picard (http://broadinstitute.github.io/picard/). Variant identification was performed using the Genome Analysis Toolkit package following the recommended best practices, including base recalibration, variant calling with Haplotype Caller, variant quality score recalibration, and variant annotation using the ANNOVAR software. Candidate causative variants that matched the following criteria were selected: (i) frequency <1% in public human databases (1000 Genomes mutation database and gnomAD); (ii) predicted to be deleterious variants according to multiple bioinformatics tools (SIFT, PolyPhen-2 and MutationTaster); (iii) bi-allelic gene variants were prioritized; and (iv) relevance for the infertile phenotype using comprehensive expression data (testis-enriched) (http://www.proteinatlas.org/) and model organism data (http://www.informatics.jax.org/mgihome/homepages/).

The *SHOC1* variants identified by WES were further validated by Sanger sequencing. PCR was conducted, and the primers used are listed in Supplementary Table S1. Subsequently, the PCR products were subjected to bidirectional Sanger sequencing using a 3730xl DNA Analyzer (Applied Biosystems, Foster City, California, USA) in Tsingke Life Technologies Biotechnology Co., Ltd. (Tsingke, Beijing, China).

### Mice

The animal experiments were approved by the Experimental Animal Management and Ethics Committee (Approval No. 2022AWS0287) and complied with the guidelines of the Animal Care and Use of Shanghai General Hospital, Shanghai Jiao Tong University School of Medicine. *Shoc1* KI mice, corresponding to the *SHOC1* variant (c.A1769G) identified in the patient (P21226), were generated using CRISPR/Cas9 genome editing technology. Briefly, Cas9 messenger RNA (mRNA), single-guide RNAs (sgRNAs), and oligonucleotides were co-injected into the zygotes of C57BL/6 mice, followed by embryo transfer into pseudopregnant ICR females. Genomic DNA was extracted from the tails of founder mice, and genotyping was performed by PCR and Sanger sequencing. The founder mice carrying the target point mutation were crossed with wild-type (WT) C57BL/6 mice to obtain offspring. PCR assays and Sanger sequencing were used to identify the point mutation in founder mice. The sequences of sgRNAs, donor oligo and the primers for genotyping are shown in Supplementary Table S1.

### Histological analysis and immunofluorescence staining

Testes, epididymis, and ovaries were detached and immediately fixed overnight in 4% paraformaldehyde. For histological analysis, the sections were processed and stained with hematoxylin and eosin (H&E) according to standard protocols as described previously [30]. For immunostaining of the sections, the tissue sections were dewaxed in xylene, rehydrated in a descending alcohol gradient, and heated in sodium citrate buffer (90°C–98°C) for 15 min for antigen retrieval. After blocking with 5% normal donkey serum (017-000-121, Jackson) for 1 h at room temperature (RT), the sections were incubated overnight with primary antibodies at 4°C. The sections were washed three times with phosphate-buffered saline containing Tween-20 (PBST) and incubated with highly cross-adsorbed secondary antibodies conjugated with Alexa Fluor^®^ 488 or Alexa Fluor^®^ 594 for 1 h at RT. The sections subsequently were washed three times with PBST and counterstained with DAPI (H-1200, Vector) to label the nuclei. Sections of the samples were imaged on an FV3000 confocal microscope (Olympus). The details of primary and secondary antibodies used are listed in Supplementary Table S2.

### Plasmid construction and mutagenesis

Total RNA was extracted from testes, HEK293T and HeLa cells using TRIzol reagent (15596026; Thermo Fisher Scientific) according to the manufacturer’s instructions and was reverse-transcribed to generate complementary DNA (cDNA) with SuperScript IV Reverse Transcriptase (6215A, TaKaRa). The cDNAs encoding all included proteins were cloned by PCR amplification from the human or mouse testis cDNA library with the PrimeSTAR system (R045A, TaKaRa). The sequences were inserted into plasmids via HR using a ClonExpress MultiS One Step Cloning Kit (C113, Vazyme). All sequences cloned into vectors were fully sequenced and then analyzed using BLAST to confirm the correct insertion of the sequences. All fusion proteins were designed to prevent the generation of frameshift-mutant proteins. Primers for constructing plasmids in this study are listed in Supplementary Table S1.

The F1–F4 truncations of SHOC1 were designed on the basis of its known functional domains and previously described regions essential for protein interactions [31, 32]. The C-terminal XPF-like domain of SHOC1, which is responsible for DNA recognition and is required for CO formation, was designated as fragment F3. The N-terminal region of SHOC1 was subdivided into two equal segments (F1 and F2). And the remaining C-terminal region beyond the XPF-like domain was defined as F4. All truncated SHOC1 plasmids (human and mSHOC1 ΔF1–ΔF4) used in this study were generated by Tsingke Life Technologies Biotechnology Co., Ltd. (Tsingke, Beijing, China).

### Cell culture and transfection

HEK293T or HeLa cells were cultured in Dulbecco’s modified Eagle’s medium (SH30243.FS, Cytiva) supplemented with 10% fetal bovine serum (FBS) (10099141C, Gibco) and 1% penicillin-streptomycin (15140122, Gibco) at 37°C in 5% CO_2_. For each well of a six-well plate, 2.5 μg of plasmids and 5 μl of Lipofectamine 3000 (L3000015, Invitrogen) were used for transfection. Cells were collected 48 h after transfection for further analysis.

### Minigene assay

The variant of *SHOC1* (c.2738–1G > A) is located at the splice-site acceptor of exon 21. We obtained the [Intron (179 bp)–Exon 21 (118 bp)–Intron (112 bp)] region by PCR and cloned it into a modified pcMINI plasmid. The variant (c.2738–1G > A) plasmid was generated by PCR with WT plasmid as the template. The WT and mutant plasmids were transfected into HEK293T cells. After culture for 48 h, total RNA was extracted using TRIzol and reverse-transcribed to obtain cDNA. RT-PCR products were separated by electrophoresis on 2% agarose gels containing ethidium bromide and visualized by exposure to UV light. Each DNA band was gel-extracted with using a gel extraction kit (D2500, Omega) and sequenced.

### Modeling of SHOC1/D-loop complex structure through molecular docking

The SHOC1/D-loop complexes were modeled using the HDOCK server [33]. Structural model prediction of WT and mutant (p.Q590R in human and p.Q646R in mice) SHOC1 proteins was performed with AlphaFold3 (https://golgi.sandbox.google.com/). The structure of D-loop was obtained from a previously solved structure of RecA-D-loop complexes (PDB ID: 7JY7) [34] and used for this docking study. The default docking parameters provided by HDOCK server were used, and the docking conformations derived from 100 top-ranked models per group were further analyzed. All structures of figures were determined using UCSF Chimera (https://www.rbvi.ucsf.edu/chimerax).

### Co-immunoprecipitation

The testicular tissue or cells were lysed in IP lysis buffer (20 mM HEPES, 150 mM NaCl, 0.5% NP-40, 1 mM DTT, pH = 7.3) supplemented with 1 × protease inhibitor cocktail (11836153001, Roche) for 30 min on ice. The lysates were clarified by centrifugation at 12 000 × *g* for 20 min at 4°C. Next, 10% of the supernatants were kept to be used as the input control, and the remainder was incubated with relevant antibodies overnight on a shaker. The lysates (500–1000 μg) were then incubated with either FLAG-beads (F2426, Sigma–Aldrich) or protein A/G magnetic beads (88803, Life Technologies) under rotation at 4°C for 2 h. The proteins associated with the protein A/G magnetic beads were collected by placing the tubes into a magnetic stand and rinsed five times with immunoprecipitation washing buffer [10 mM Tris–HCl, pH 7.5, 1 mM Ethylenediaminetetraacetic acid (EDTA), 150 mM NaCl, and 0.1% Triton X-100], after which they were subjected to subsequent analysis.

### Western blot

The testicular tissue or cells were rinsed with PBS and lysed in RIPA lysis buffer (89901, Sigma-Aldrich) supplemented with 1 × protease inhibitor cocktail (11836153001, Roche) for 30 min on ice. Following centrifugation at 12 000 × *g* for 20 min at 4°C, the protein concentration of the lysates was determined using a BCA kit (23225, Thermo Fisher Scientific). Sodium dodecyl sulfate–polyacrylamide gel electrophoresis (SDS–PAGE) was performed using 20 μg of lysate from each sample, and Western blot (WB) was performed as previously described [35]. After SDS‒PAGE was performed on a 10% gel at 80 V for 30 min followed by 120 V for 80 min to load and separate the protein samples, the samples were transferred to a 0.22 μm PVDF membrane (ISEQ00010, Millipore). The membranes were then blocked with 5% skim milk and incubated with the primary antibodies overnight at 4°C. The next day, the membranes were washed three times with TBS containing 0.1% Tween (TBST) and then incubated with horseradish peroxidase-conjugated secondary antibodies for 1 h at RT. After three washes in TBST, the blots were detected by chemiluminescence (WBKLS0500, Millipore). Densitometry data were analyzed using the AI600 software. The housekeeping protein *β*-actin was used for WB normalization. The details of primary and secondary antibodies are listed in Supplementary Table S2.

### Spermatocyte spreads and immunostaining

Mouse testicular cells were prepared for surface spreading and subsequent immunostaining as previously described [30] with the following modifications. The testicular tissue was macerated in PBS, followed by removal of the tunica albuginea. The seminiferous tubules were incubated in hypotonic extraction buffer (0.6 M Tris, pH 8.2, 500 μl; 0.5 M sucrose, 1 ml; 0.17 M trisodium citrate dihydrate, 1 ml; 0.5 M EDTA, pH 8.0, 100 μl; 0.5 M DTT, 50 μl; 0.1 M phenylmethanesulfonyl fluoride (PMSF), 100 μl; ddH_2_O, 7.25 ml) for 25 min at RT. Subsequently, the cells were centrifuged and resuspended in 100 mM sucrose and spread on slides with 1% PFA (pH 9.2) containing 0.15% Triton X-100. Slides were then placed in a humidified chamber for at least 3 h. Last, slides were washed twice for 3 min in 0.4% Photoflo (Kodak) and air-dried at RT. Slides were either used for immunostaining immediately or stored at −80°C. The immunostaining procedure was conducted as described earlier, and the details of primary and secondary antibodies are listed in Supplementary Table S2. Spermatocytes were staged on the basis of the development of axial/lateral elements (AEs/LEs, labeled by SYCP3) and chromosome synapsis (marked by SYCP1 or SIX6OS1 signals), as previously described [23]. Leptotene: short stretches of AEs without synapsis; early zygotene: relatively long and incomplete LEs with visible SIX6OS1 threads; late zygotene: intact LEs but incomplete chromosome synapsis; early pachytene: all autosomes have synapsed, and thickness of the synapsed axes is uniform, synapsed pseudoautosomal region (PAR) is long (length is >3 × the widths); mid-pachytene: the morphology of the SC is similar to that in early pachytene, synapsed PAR is short (length is <3 × the widths), and the X chromosome also becomes shorter than that in early pachytene; late pachytene: the ends of synapsed autosomes are thicker than the interstitial regions, synapsed PAR is very short, dot-like, or with a small gap between them; diplotene: central elements (CEs) start to disassemble, but the LEs remain intact, the X chromosome is relatively long and curved. To stage the spermatocytes in *Shoc1* KI mice, the same criteria as those used for control mice were applied for the leptotene and early zygotene. Due to synaptic defects in spermatocytes of *Shoc1* mutant mice, subsequent substages were classified on the basis of the length and thickness of the LEs of SCs and morphology of the sex chromosomes.

### Bimolecular fluorescence complementation assay

The bimolecular fluorescence complementation (Bi-FC) assay was performed using Venus fragments, which detects fluorescence generated by complementation between non-fluorescent N- and C-terminal fragments fused to interacting proteins as previously described [36]. On the basis of the Venus system (Addgene), we fused VN210 (Venus aa1-210) to human/mSHOC1 and TEX11 (generating SHOC1-nVenus and TEX11-nVenus), and VC210 (Venus aa210-238) to human/mouse TEX11 and C1orf146/SPO16 (generating TEX11-cVenus and C1orf146/SPO16-cVenus). These constructs were transfected into HeLa cells, with protein–protein interactions visualized by fluorescence imaging.

### Single-cell RNA sequencing analysis

Samples from adult *Shoc1* KI and littermate control mice were used for single-cell RNA sequencing (scRNA-seq). Detailed scRNA-seq analysis of testicular samples has been described in the previous study [37]. Briefly, individual mouse testicular cells were obtained through enzymatic digestion and loaded on a 10× Genomics chip to form emulsion gel beads, which were subjected to reverse transcription and PCR amplification, and then sequenced on an Illumina NovaSeq 6000. A cell-gene expression matrix was constructed with Cell Ranger software (10× Genomics). After quality control, standardization, and dimensionality reduction, cell identification and clustering analysis were performed in the R environment. Clustering was performed using Seurat (V4.1.1) R package on 30 principal components. Following initial clustering, cell subpopulations were annotated in accordance with previously established marker genes [38–40]. Briefly, *Ddx4* and *Vim* were used as markers to distinguish germ cells and somatic cells, respectively. Within the germ cell population, spermatogonial stem cells were identified using *Utf1, Gfra1*, and *Uchl1*, while differentiating spermatogonia were marked by *Kit* and *Stra8*. Spermatocytes were characterized by *Sycp1, Spo11, Piwil1* and *Ovol2*, allowing further classification into sub-stages of meiosis I—leptotene, zygotene, pachytene, and diplotene—as well as meiosis II cells. Finally, spermatids and sperm were identified on the basis of the expression of *Tnp1* and *Prm2*. The Seurat *FindMarkers* function was used for identification of differentially expressed genes (DEGs) in the *Shoc1* KI group versus control pachytene cluster group. Gene Ontology (GO) biological processes, Kyoto Encyclopedia of Genes and Genomes pathways, and gene set enrichment analysis (GSEA) were performed using WebGestalt 2024 (https://www.webgestalt.org).

### Purification of pachytene spermatocytes

The modified STA-PUT velocity sedimentation was used for purification of pachytene spermatocytes from mouse testicular tissues as previously reported [37, 41]. To obtain testicular cell suspensions, mouse testes were enzymatically digested with 4 mg/ml collagenase type IV (17104-019, Life Technologies), 2.5 mg/ml hyaluronidase (H3506, Sigma–Aldrich), and 1 mg/ml trypsin (T8003, Sigma–Aldrich) at 37°C for 20 min. Next, specific cell populations were enriched by gravity sedimentation through a discontinuous bovine serum albumin density gradient. The cell fractions were then manually collected and the purity of different types of cells was measured by immunofluorescence (IF) staining and morphology. Each purification was performed on testes from four mice. The spermatocyte samples with a purity of>75% were used for subsequent sequencing.

### Hi-C library construction

Briefly, isolated cells were fixed with 2% formaldehyde at RT for 10 min. Then formaldehyde was quenched with glycine for 10 min at RT. Cells were washed with 1 × PBS twice and then lysed in 50 μl lysis buffer (10 mM Tris-HCl, pH 7.4, 10 mM NaCl, 0.1 mM EDTA, 0.5% NP-40, and proteinase inhibitor cocktail) on ice for 50 min. After centrifugation at 3000 rpm for 5 min at 4°C, the supernatant was carefully discarded with a pipette. Chromatin was solubilized in 0.5% SDS and incubated at 62°C for 10 min. SDS was quenched by 10% Triton X-100 at 37°C for 30 min. Then, the nuclei were digested with 50 U of DpnII at 37°C overnight with rotation. DpnII was then inactivated at 62°C for 20 min. To incorporate biotin-labeled dCTP into DNA, dATP, dGTP, dTTP, biotin-14-dCTP, and Klenow were added to the solution, and the reaction was carried out at 37°C for 1.5 h with rotation. The fragments were ligated at RT for 6 h with rotation. This was followed by reversal of crosslinking and DNA purification. DNA was sheared to 300–500 bp with Covaris M220. The biotin-labeled DNA was then pulled down with 10 μl of Dynabeads MyOne Streptavidin C1 (Invitrogen, 65001). Sequencing library preparation was performed on beads, including end-repair, dATP tailing, and adaptor-ligation, was performed on the beads. DNA was eluted twice by adding 20 μl water to the tube and incubating it at 66°C for 20 min. Nine to 15 cycles of PCR amplification were performed with Extaq (Takara, RR001). Finally, size selection was performed with AMPure XP beads, and fragments ranging from 200 to 1000 bp were selected. The library was sequenced on MGI DNBSEQ T7 according to the manufacturer’s instructions.

### Hi-C data analysis

We obtained a WT Hi-C dataset from mouse cells at the pachytene stage from published work (accession no. GSE109344) [42]. FASTQ files of Hi-C libraries were processed by the Juicer pipeline with default settings (v1.6) [43], and the mm10 reference genome was used for mapping. The obtained .hic files were converted into cool format using the hic2cool package for subsequent downstream analyses. The *cis* and trans expected contact probabilities were calculated using the cooltools package (v0.7.0) [44]. Contact probability (P(s)) curves were computed from the cool files binned at 5-kb resolution using cooltools.expected_*cis* from Cooltools. For compartment analysis, eigenvector decomposition was performed at a resolution of 500 kb using the eigenvector function in Juicer. The topologically associated domain (TAD) insulation score was calculated at 25-kb resolution by Cooltools. The pileups of 500-kb genomic regions flanking the TAD boundaries were performed at 25-kb resolution using coolpup.py under the local mode (https://github.com/open2c/coolpuppy, v1.1.0) [45]. The identification of COs–DSBs regions followed the methods described in the previously published study [46]. Specifically, the positions of DSB hotspots were obtained from the previously published DMC1 ChIP-Seq data [47] and converted into 10-kb genomic bins. The positions of CO hotspots were obtained from the single-sperm DNA sequencing data [48]. The 10-kb genomic bins containing DSBs were designated as COs–DSBs if they overlapped with CO hotspots. Pileup heatmaps for 2-Mb genomic regions centered at the selected COs–DSBs regions were generated at a resolution of 5 kb. The observed/expected (O/E) interaction values were extracted and statistically summarized in Python to compare the chromatin interactions around COs–DSBs.

### Statistical analysis

The data are shown as the mean ± SEM unless otherwise indicated. Statistical significance was determined using the Student’s *t*-test (for the results in Figs 3M–O, 4B, D, F, H, J, L, O–R, and 6F; Supplementary Figs S3B, S5B, S6B, S7D, S8B, D, F, H, S9E, J, and S11B, D; unpaired, two-tailed), or one-way analysis of variance (ANOVA) of variance (for the results in Fig. 3D) with GraphPad Prism 9 software. The confidence interval was 95%. Statistical significance was determined using the Student’s *t*-test (for the results in Figs 5M and 7G; unpaired, two-tailed) with R software (version 4.3.1; R Foundation for Statistical Computing, Vienna, Austria). *P* < .05 was considered to indicate statistical significance. Statistical parameters are reported in the figures or their legends.

## Results

### Human SHOC1 interacted with M1AP, REDIC1, and ZZS protein TEX11, but not C1orf146 (an ortholog of SPO16) *in vitro*

**Figure 1.**
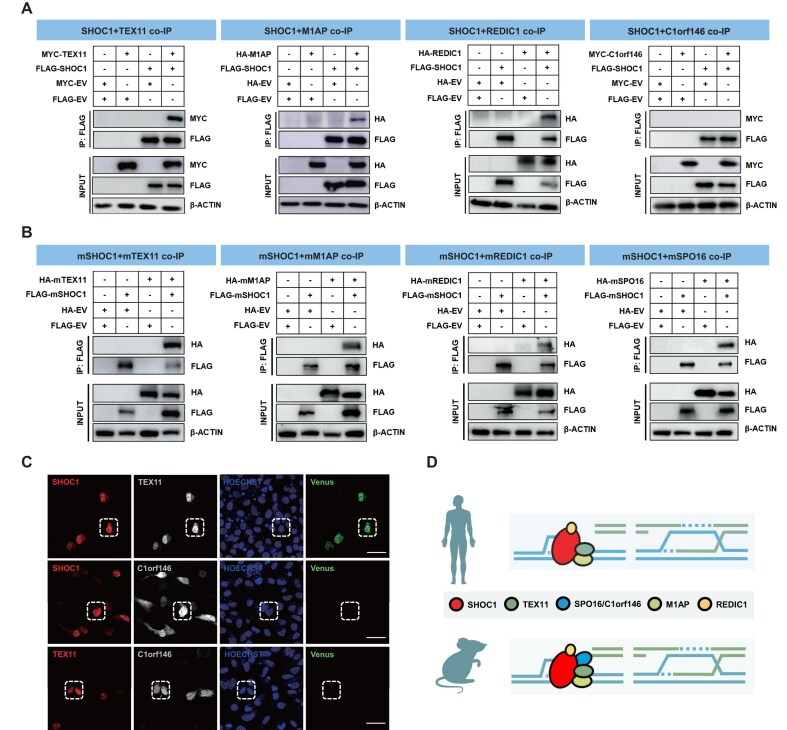
Distinct models for human and mSHOC1 complexes by *in vitro* analysis. (**A**) Co-IP demonstrated the interaction of human SHOC1 (detected via N-terminal FLAG tag) with M1AP, REDIC1, and ZZS protein TEX11, but not with C1orf146 (detected via N-terminal HA or MYC tag). Input lysates were included. (**B**) Co-IP confirmed the interaction of mSHOC1 (detected via N-terminal FLAG tag) with mM1AP, mREDIC1, and ZZS proteins mTEX11 and mSPO16 (detected via N-terminal HA tag). Input lysates were included. (**C**) Bi-FC revealed Venus fluorescence signals in human SHOC1- and TEX11-coexpressing cells, but not in SHOC1/C1orf146- or TEX11/C1orf146-coexpressing groups. Scale bars, 50 μm. (**D**) Schematic illustration of the SHOC1 complex components in human and mouse.

To determine the components of SHOC1 complex in humans, we performed co-immunoprecipitation (co-IP) assay. Human FLAG-tagged SHOC1 was co-expressed with human HA-tagged M1AP or REDIC1, or MYC-tagged TEX11 or C1orf146 in HEK293T cells. Proteins were immunoprecipitated from cell lysates by tag-specific antibodies. Subsequent WB analysis revealed that human SHOC1 binds specifically with M1AP, REDIC1, and ZZS proteins of TEX11. However, we did not detect a specific interaction between human SHOC1 and C1orf146 *in vitro*, which differs from the previous report in mice [13] (Fig. 1A and B). Specifically, mSHOC1 could interact with all partners, including TEX11, M1AP, REDIC1, and SPO16.

In addition, the interaction between mouse TEX11 and SPO16 was consistently detected regardless of the presence or absence of mSHOC1, suggesting that mouse TEX11–SPO16–SHOC1 complex may form a stable, pre-assembled functional module (Supplementary Fig. S1A). In contrast, no interaction was observed between human TEX11 and C1orf146 even when human SHOC1 was co-expressed (Supplementary Fig. S1B). Furthermore, Bi-FC assays confirmed these interaction dynamics: while mSHOC1, TEX11, and SPO16 clearly exhibited clear Venus fluorescence signals in all pairwise combinations (SHOC1/TEX11, SHOC1/SPO16, and TEX11/SPO16) (Supplementary Fig. S1C), human Venus signals were detected only in the SHOC1/TEX11 co-expressing group (Fig. 1C). Thus, we proposed distinct models for the SHOC1 complex in humans and mice *in vitro* (Fig. 1D).

To further determine the specific region of SHOC1 involved in interactions with other partners, we co-expressed M1AP, REDIC1, or TEX11 full-length proteins and various SHOC1 truncated proteins (Human: N-terminal 1–576, C-terminal 577–1444, ΔF1 aa1-288del, ΔF2 aa289-576del, ΔF3 aa577-1098del, ΔF4 aa1099-1444del; Mouse: N-terminal 1–625, C-terminal 626–1482, ΔF1 aa1-312del, ΔF2 aa313-625del, ΔF3 aa626-1154del, ΔF4 aa1155-1482del) in HEK293T cells (Supplementary Fig. S2A and B). Co-IP and subsequent WB analysis demonstrated that truncation of specific regions (ΔF1–ΔF4) of SHOC1 did not disrupt the binding of M1AP or REDIC1 (Supplementary Fig. S2C and D). However, removal of the N-terminal region of SHOC1 completely abolished TEX11 binding in both mouse and human, suggesting that the isolated SHOC1 N-terminal region is essential for interaction with TEX11. Interestingly, ΔF1 and ΔF2 fragments of mSHOC1 did not affect TEX11 binding, indicating the presence of TEX11 binding sites in both fragments. In contrast, ΔF1 and ΔF2 fragments in human SHOC1 completely abolished the TEX11 binding, underscoring the necessity of an intact N-terminal region for maintaining the SHOC1–TEX11 interaction in humans (Supplementary Fig. S2C and D). These findings further suggested that there are differences in the interaction patterns between human and mSHOC1 and its partners, particularly with respect to TEX11, which indicates a potential species-specific function of human SHOC1 in meiosis that warrants further investigation.

### Missense variant within the XPF-like domain in SHOC1 (p.Q590R) was associated with meiotic arrest and NOA

**Figure 2.**
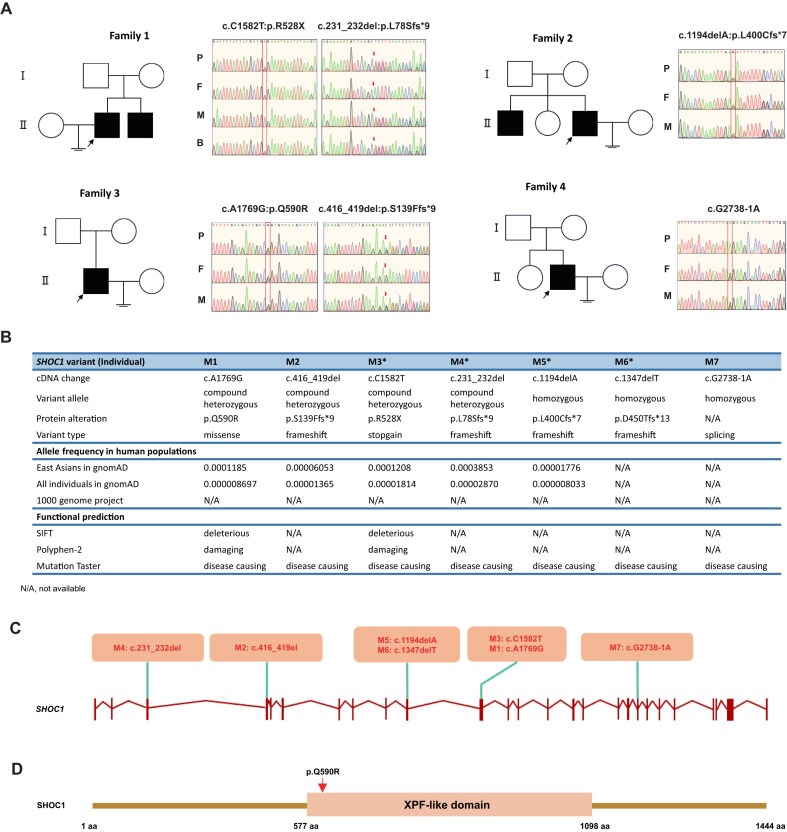
Bi-allelic *SHOC1* variants were identified in NOA-affected patients with meiotic arrest. (**A**) Pedigrees of infertile families carrying *SHOC1* variants. WES findings were validated by Sanger sequencing. Probands are indicated by black arrows, and variant-affected nucleotides are highlighted with red dash line boxes or arrows. (**B**) Summary of *SHOC1* variants identified in this study, with asterisks denoting variants reported in our previous publication. (**C**) The localization of identified *SHOC1* variant residues. (**D**) Schematic diagram of the human SHOC1 protein, annotated with functional domains. The p.Q590R variant is highlighted by a red arrow.

Idiopathic NOA caused by meiotic arrest is a common cause of male infertility and has many genetic origins. Here, we screened for potential variants in a cohort of 171 patients with meiotic arrest and found bi-allelic *SHOC1* variants were associated with NOA. In addition to the compound heterozygous variants in *SHOC1* (M3: c.C1582T:p.R528X and M4: c.231_232del:p.L78Sfs*9) and homozygous loss-of-function (LoF) variants (M5: c.1194delA:p.L400Cfs*7 and M6: c.1347delT:p.D450Tfs*13) in our previous work [30], novel compound heterozygous variants (M1: c.A1769G:p.Q590R and M2: c.416_419del:p.S139Ffs*9) in family 3 and a homozygous splicing variant (M7: c.G2738-1A) of *SHOC1* in family 4 were identified after the genetic analysis pipeline in the current study. All variants were absent or had low allele frequencies in public databases. Sanger sequencing validated all above *SHOC1* variants in pedigrees affected by NOA, consistent with autosomal recessive inheritance patterns following pedigree co-segregation analysis (Fig. 2). Clinical details for each patient are outlined in Supplementary Table S3.

All the *SHOC1* variants identified in this study included one missense (M1), four frameshifts (M2, M4, M5, and M6), one stop-gain (M3), and one splicing (M7) type. Among them, the variant M2–M6 variants were predicted to result in the deletion of most of the regions of SHOC1 protein and were assumed to be pathogenic on the basis of the results of pathogenicity prediction tools, including SIFT, PolyPhen-2, and MutationTaster (Fig. 2B). Next, we transfected the full-length WT and mutant cDNA constructs of *SHOC1* into HEK293T cells. As a result, we observed protein degradation in the M2 and M4 variants and truncations in the M3, M5, and M6 variants, whereas the M1 variant had no effect on protein expression, displaying levels comparable to those of the control group. (Supplementary Fig. S3A and B). Therefore, we proposed that all LoF variants (M2–M6) in *SHOC1* were potential disease-causing and associated with male infertility in the affected individuals. The variant (M7) was located in the region of the canonical splice donor site at the boundary of exon 21. To assess the impact of the variant on *SHOC1* splicing, minigene vectors containing the genomic sequence spanning exon 21 and flanking introns of the *SHOC1* gene were transfected into HEK293T cells, followed by RT-PCR. The WT SHOC1 minigene showed a canonical splicing pattern, while the splicing variant M7 resulted in a band smaller than that of WT (Supplementary Fig. S3C). Sanger sequencing revealed that the splicing variant caused skipping of exon 21 (Supplementary Fig. S3D), which was assumed to result in the production of truncated proteins deficient in functional segments within the XPF-like domain. This result suggested that the splicing variant M7 in *SHOC1* (c.G2738-1A) induced aberrant mRNA splicing (Supplementary Fig. S3E).

The only identified missense variant M1 in family 3, which is located within the functional XPF-like domain, was also predicted to be pathogenic by all three tools (Fig. 2B), underscoring the importance of this residue within the XPF-like domain in spermatogenesis. Testicular biopsies of the proband harboring the compound heterozygous variants (M1: c.A1769G:p.Q590R and M2: c.416_419del:p.S139Ffs*9) of *SHOC1* in family 3 were obtained and the H&E staining revealed that all analyzed seminiferous tubules contained spermatogonia and spermatocytes but lacked spermatids and spermatozoa. In contrast, the testicular histopathology of testes from OA-affected patients revealed normal spermatogenesis (Supplementary Fig. S4A). Moreover, IF staining and meiotic chromosomal spread analysis through various marker combinations (SYCP3 & γH2AX; DMC1 & PNA) also verified the meiotic arrest phenotype in this proband (Supplementary Fig. S4B–D). Collectively, these findings underscore a critical role of the XPF-like domain in SHOC1, especially the Q590 residue in human meiosis, and establish one clinical-genetic paradigm for mechanistic dissection of male infertility caused by the missense variant M1 within the XPF-like domain (Supplementary Fig. S4E).

### *Shoc1* mutation (p.Q646R) resulted in sterility in both male and female mice

**Figure 3.**
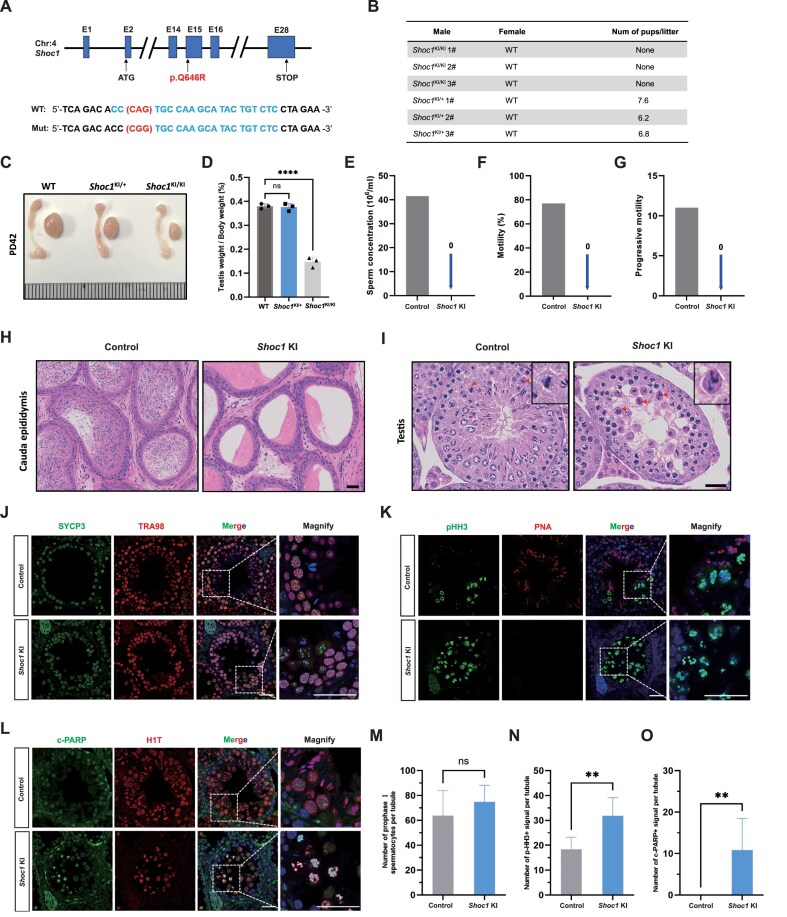
Male mice homozygous for the *Shoc1* missense variant (p.Q646R) exhibited spermatogenic failure and MMI arrest. (**A**) CRISPR/Cas9-mediated genome engineering strategy used to generate a C57BL/6J mouse model carrying the *Shoc1* mutation (p.Q646R). (**B**) Fertility assessment of *Shoc1* KI homozygous (*Shoc1*^KI/KI^) mice and littermate controls (*Shoc1*^KI/+^). (**C**) Representative image comparing testis size in adult *Shoc1* KI homozygous (*Shoc1*^KI/KI^) mice and littermate controls (*Shoc1*^KI/+^ and WT). (**D**) Testis-to-body weight ratios quantified for *Shoc1* KI homozygous (*Shoc1*^KI/KI^) mice and littermate controls (*Shoc1*^KI/+^ and WT) using one-way ANOVA; **** *P* < .0001; ns, not significant; error bars, mean ± SEM. (**E**–**G**) Epididymal sperm count and motility were analyzed by CASA. H&E-stained epididymal (**H**) and testicular (**I**) sections from adult *Shoc1* KI homozygous (*Shoc1*^KI/KI^) mice and littermate controls (*Shoc1*^KI/+^). Scale bars, 50 μm. (**J**) IF staining of SYCP3 (green) and TRA98 (red) in testicular sections from adult *Shoc1* KI homozygous (*Shoc1*^KI/KI^) mice and littermate controls (*Shoc1*^KI/+^). Scale bars, 50 μm. (**K**) IF staining of pHH3 (green) and PNA (red) in testicular sections from adult *Shoc1* KI homozygous (*Shoc1*^KI/KI^) mice and littermate controls (*Shoc1*^KI/+^). Scale bars, 50 μm. (**L**) IF staining of c-PARP (green) and H1T (red) in testicular sections from adult *Shoc1* KI homozygous (*Shoc1*^KI/KI^) mice and littermate controls (*Shoc1*^KI/+^). Scale bars, 50 μm. Quantification of SYCP3 (**M**), pHH3 (**N**), and c-PARP (**O**) positive signals in testicular sections from adult *Shoc1* KI homozygous (*Shoc1*^KI/KI^) mice and littermate controls (*Shoc1*^KI/+^) using two-tailed Student’s *t*-test; ** *P* < .01; ns, not significant; error bars, mean ± SEM.

To determine the specific role of the XPF-like domain in SHOC1 during meiosis, we applied CRISPR/Cas9-mediated gene targeting to generate *Shoc1* mutant (p.Q646R) KI (*Shoc1* KI) mice, which mimic the identified missense variant M1 (p.Q590R) within the XPF-like domain in *SHOC1* in family 3 (Fig. 3A). Subsequent assessment of fertility in adult *Shoc1* KI mice revealed complete sterility in all tested homozygous males (Fig. 3B). Furthermore, smaller ovaries and follicular dysplasia were detected in adult *Shoc1* KI females (Supplementary Fig. S5). Additionally, compared with their littermate controls, adult KI male mice displayed significantly smaller testes and lower testis-to-body weight ratio (Fig. 3C and D). Computer-aided sperm analysis (CASA) showed the absence of sperm in the cauda epididymis of adult *Shoc1* KI mice (Fig. 3E–G). Histological analysis demonstrated a complete lack of post-meiotic germ cells in all seminiferous tubules of the testes, with no mature sperm present in the cauda epididymis in adult *Shoc1* KI mice. In contrast, the control group exhibited abundant germ cells at various developmental stages in the testes and mature sperm in the cauda epididymis (Fig. 3H and I). Notably, many MMⅠ spermatocytes with condensed nuclei and misaligned chromosomes were found in KI mice (Fig. 3I).

IF analysis of testicular sections from adult male mice revealed spermatogenic failure in the KI group compared to controls. A significant reduction in TRA98-positive germ cells was observed in the KI group (Fig. 3J). Intriguingly, semi-quantitative assessment revealed that the number of prophase I spermatocytes remained comparable between groups (Fig. 3J and M). To clarify the exact substage of meiotic defects that occurred in *Shoc1* KI males, we further examined the progression of meiotic prophase I by immunostaining spermatocyte spreads for SYCP3 and γH2AX (a marker of DNA breaks). Distinct proportions of leptotene, zygotene, pachytene, and diplotene spermatocytes were observed between control and KI mice. Notably, a distinct population of cells—accounting for 41.9% of total spermatocytes—was identified exclusively in KI testes. These cells morphologically resembled pachytene spermatocytes but exhibited aberrant γH2AX signals on autosomes and are referred to in this study as pachytene-like spermatocytes (Supplementary Fig. S6). Mechanistically, this stable population size results from the failure to activate pachytene checkpoints, allowing defective cells to bypass early meiotic surveillance. We found that no appreciable difference was observed in the recruitment of BRCA1 (a critical mediator for the asynapsis checkpoint) along autosomal axes outside the sex body between *Shoc1* KI and control spermatocytes. (Supplementary Fig. S7A). The DNA damage checkpoint effector p-CHK1 did not exhibit a significant increase during the pachytene-like stage (Supplementary Fig. S7B), indicating that these cells escape pachytene checkpoint-mediated elimination during prophase I.

Notably, the KI group exhibited complete depletion of post-meiotic haploid spermatids identified by PNA labeling, accompanied by a significant accumulation of pHH3-positive MMI spermatocytes (Fig. 3K and N). Immunostaining revealed a nearly twofold increase in BUB3-positive spermatocytes in *Shoc1* KI mice compared with control mice (Supplementary Fig. S7C and D), indicating that this developmental arrest is driven by the persistent activation of the spindle assembly checkpoint (SAC). An increase in c-PARP signaling indicated increased apoptosis in *Shoc1* KI seminiferous tubules (Fig. 3L and O). Combined with cytological identification of condensed bivalent chromosomes and pHH3-positive signals, these results revealed that these apoptotic events were specifically localized to arrested MMI spermatocytes. These findings collectively demonstrated that *Shoc1* KI induced meiotic arrest at MMI, characterized by activation of the SAC but not the pachytene checkpoint.

### The Q646 residue within the XPF-like domain in SHOC1 is required for CO formation by orchestrating SHOC1 complex assembly during meiotic recombination

**Figure 4.**
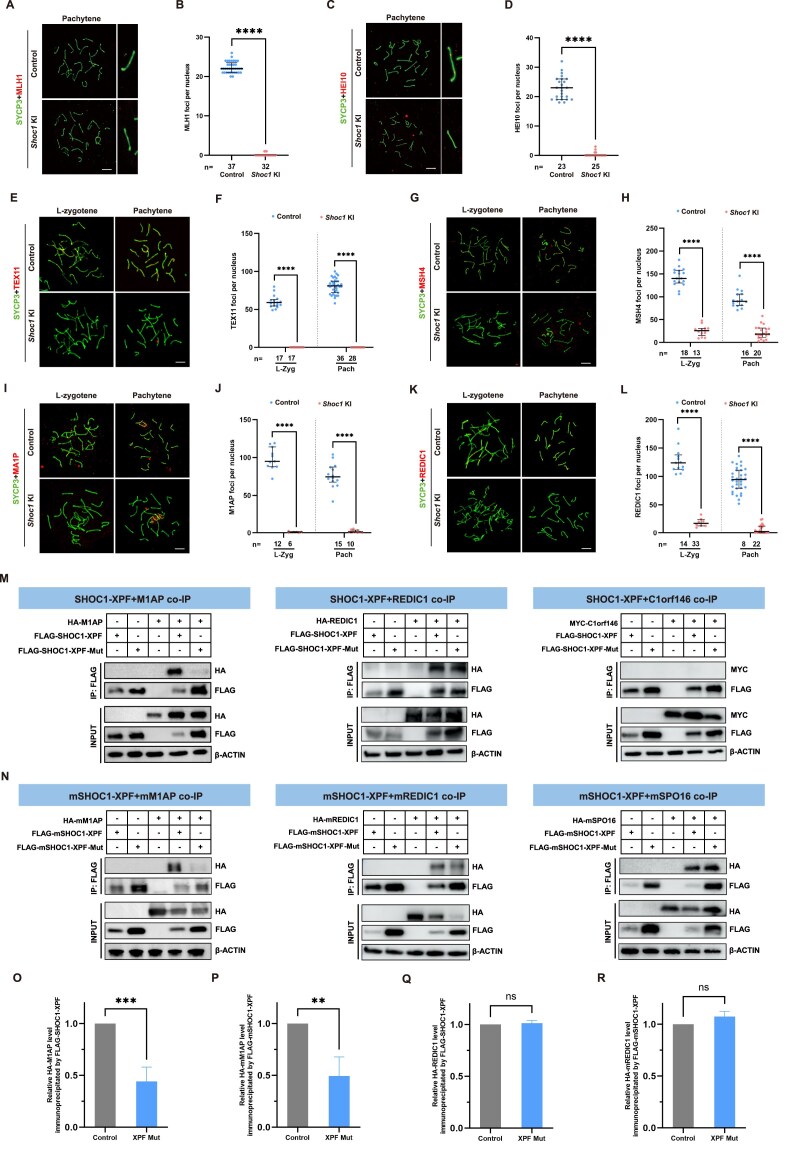
*Shoc1* KI mice exhibited defective CO formation and disrupted dynamics of meiotic recombination intermediates. Representative images of spread spermatocytes from adult *Shoc1* KI homozygous (*Shoc1*^KI/KI^) mice and littermate controls (*Shoc1*^KI/+^) co-stained with SYCP3 (green) and MLH1 (red, **A**), HEI10 (red, **C**), TEX11 (red, **E**), MSH4 (red, **G**), M1AP (red, **I**), and REDIC1 (red, **K**). Scale bars, 10 μm. Quantification of MLH1 (**B**), HEI10 (**D**), TEX11 (**F**), MSH4 (**H**), M1AP (**J**), REDIC1 (**L**) foci per cell at indicated meiotic stages using two-tailed Student’s *t*-test; **** *P* < .0001; error bars, mean ± SEM; *n*, the total number of nuclei analyzed. (**M**) Co-IP assays in HEK293T cells assessing interactions between human SHOC1-XPF (WT or mutant, FLAG-tagged) and M1AP/REDIC1/C1orf146 (HA/MYC-tagged). Input lysates were included. (**N**) Co-IP assays in HEK293T cells assessing interactions between mSHOC1-XPF (WT or mutant, FLAG-tagged) and M1AP/REDIC1/SPO16 (HA/MYC-tagged). Input lysates were included. Relative interaction intensities quantified for human (**O**) or mouse (**P**) SHOC1-XPF (mutant) and M1AP / SHOC1-XPF (WT) and M1AP using two-tailed Student’s *t*-test; *** *P* < .001; ** *P* < .01; error bars, mean ± SEM. Relative interaction intensities quantified for human (**Q**) or mouse (**R**) SHOC1-XPF (mutant) and REDIC1 / SHOC1-XPF (WT) and REDIC1 using two-tailed Student’s *t*-test; ns, not significant; error bars, mean ± SEM. L-Zyg, late zygotene; Pach, pachytene.

MMI spermatocytes exhibiting chromosome misalignment often arise from defective CO formation between homologous chromosomes during prophase I [49]. Thus, we quantified the number of MLH1 foci in pachytene stage, which marked sites of class I COs, in both KI and control groups. Control spermatocytes displayed 23.0 ± 1.5 MLH1 foci per nucleus, whereas the KI group showed nearly complete absence, with only 0.1 ± 0.3 foci per nucleus (Fig. 4A and B). The HEI10 E3 ligase is another late recombination nodule component and is directed by MutLγ to stably accumulate at designated CO sites. Similarly, the number of HEI10 foci was greatly reduced in *Shoc1* KI mice (0.3 ± 0.7 versus 22.8 ± 3.9) (Fig. 4C and D). These findings confirmed that the p.Q646R mutation disrupted CO formation in KI group.

Given the known DSBs repair defects in *Shoc1*^−/−^ mice [14], we then analyzed the expression of two essential recombinases, DMC1 and RAD51. In control spermatocytes, DMC1 and RAD51 localized to ssDNA at the early-zygotene stage, and were removed from these sites during the late-zygotene to pachytene transition. Compared with those in the early-zygotene stage, the numbers of DMC1 and RAD51 in the early-pachytene stage were reduced to approximately one-tenth of those in the early-zygotene stage. In *Shoc1* KI cells, DMC1 and RAD51 were similarly recruited to ssDNA at the early-zygotene stages but gradually decreased from the early-zygotene stage to the pachytene stage. However, compared with those in the control groups, the numbers of both types of foci in early pachytene KI spermatocytes were significantly greater (Supplementary Fig. S8A–D). Similarly, the number of SPATA22 and RPA2 foci in KI cells, which are well known for binding with ssDNA overhangs during homologous recombination (HR), was also comparable to that in control cells in zygotene but greater in early-pachytene spermatocytes (Supplementary Fig. S8E–H). Altogether, these results revealed impaired DSB repair in *Shoc1* KI spermatocytes.

During DSB recombination, ZMM proteins are recruited to recombination intermediates to facilitate the generation of COs-prone joint molecules, SEIs, and double holiday junctions. In control spermatocytes, TEX11 localized to recombination sites during meiotic prophase I, specifically marking the COs-prone recombination nodules in zygotene and early-pachytene spermatocytes. In contrast, TEX11 foci were absent in *Shoc1* KI cells (Fig. 4E and F), indicating that the mutation within the XPF-like domain disrupted TEX11 localization. Additionally, MSH4, another component of the ZMM complex that facilitates the assurance and interference of COs, is localized to mid-recombination intermediates in control early-pachytene meiocytes. Notably, a reduced number of MSH4 foci was observed in zygotene and pachytene spermatocytes of *Shoc1* KI males (Fig. 4G and H), suggesting that the mutation within the XPF-like domain also influenced the recruitment of MSH4 foci to the chromosome axis and the stabilization of recombination intermediates. Intriguingly, the significantly decreased expression profiles in M1AP and REDIC1, two components of the SHOC1 complex mentioned earlier, were also detected from late zygotene to pachytene stages in *Shoc1* KI males (Fig. 4I–L). To define the interactions after the mutation within XPF-like domain, we generated FLAG-tagged SHOC1-XPF variants (p.Q646R) across species. Reciprocal co-IP assay was performed using anti-FLAG antibody, revealing reciprocal interactions between M1AP, REDIC1, SPO16, and SHOC1 XPF-like domain in mice, whereas SHOC1 XPF-like domain could interact with M1AP and REDIC1 but not SPO16 in humans. In addition, the mutant SHOC1 (whether human or mouse origin) showed a significantly weaker interaction with M1AP instead of the other two proteins (Fig. 4M–R). Taken together, the disruption of molecular interactions was associated with the destabilization of the recombination intermediates in *Shoc1* KI mice.

The XPF-like domain in SHOC1 was reported to bind with branched DNA structures, which are essential for meiotic recombination. To elucidate the effect of the interaction between branched recombination intermediates and the mutated XPF-like domain in SHOC1 (p.Q590R in humans or p.Q646R in mice), we performed molecular dynamics simulations of WT and mutant SHOC1 proteins with the branched DNA (D-loop) structure (PDB ID: 7JY7). The human and mSHOC1/D-loop complexes were modeled using the HDOCK server with high confidence scores in WT (0.933 in humans and 0.907 in mice) and KI groups (0.928 in humans and 0.957 in mice) (Supplementary Fig. S9 A, B, F, and G). Comparative analysis revealed a marked reduction in residue hydrogen bond formation mediated by human R590 (mouse R646) in the mutant complex relative to the WT configuration (Supplementary Fig. S9C, D, H, and I). This substitution replaced the uncharged polar glutamine with a positively charged arginine, fundamentally altering local electrostatic properties that may perturb protein stability and intermolecular interactions. Quantitative analysis of docking scores derived from 100 top-ranked models per group further demonstrated significantly lower binding energy for the mutant SHOC1/D-loop complex than for the WT (human: −190.6 ± 18.7 in p.Q590R versus −202.2 ± 20.9 in WT, *P* < .0001; mouse: −189.4 ± 21.7 in p.Q646R versus −203.5 ± 16.8 in WT, *P* < .0001) (Supplementary Fig. S9E and J). This substitution-induced conformational change appeared to mechanistically impair branched DNA binding ability of the XPF-like domain in SHOC1 during meiotic recombination. Thus, these data demonstrated that the XPF-like domain in SHOC1 ensures CO formation via orchestrating the assembly of the SHOC1 complex and binding to branched DNA structures.

### The disruption of dynamic 3D chromatin structure and CO region interaction in Shoc1 KI pachytene spermatocytes

**Figure 5.**
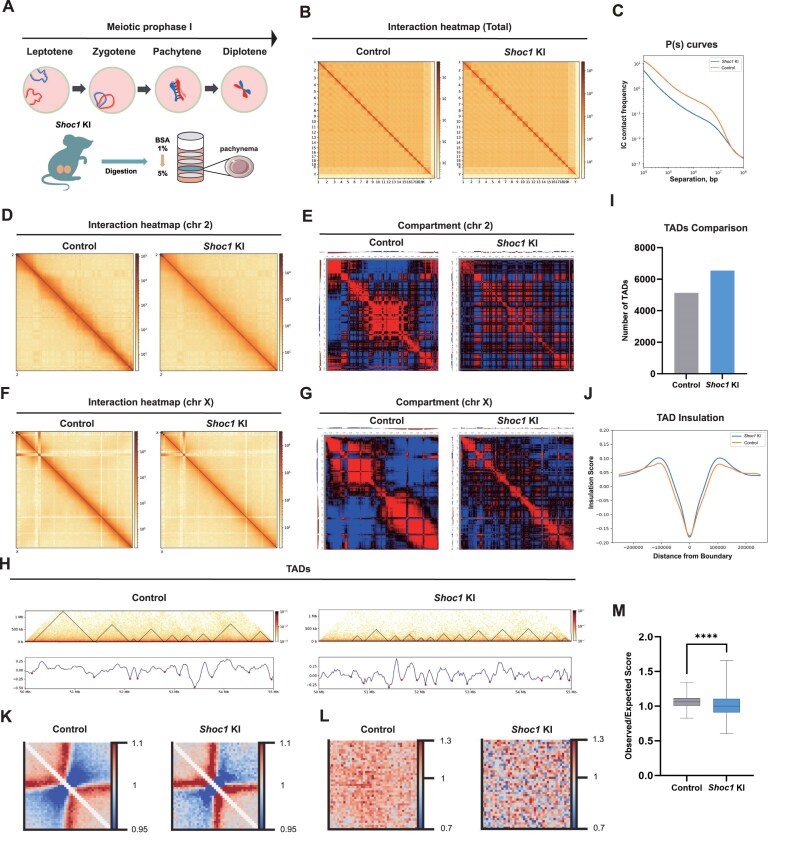
The disruption of dynamic 3D chromatin structure and CO region interaction in *Shoc1* KI pachytene spermatocytes. (**A**) Schematic of mouse meiotic prophase Ⅰ and collection of pachytene spermatocytes by STA-PUT method. (**B**) Normalized Hi-C interaction heatmaps (2.5 MB bins) for all chromosomes in pachytene spermatocytes from adult *Shoc1* KI homozygous (*Shoc1*^KI/KI^) mice and controls (WT Hi-C dataset). (**C**) Chromatin contact probabilities relative to genomic distance (P(s) curves) between pachytene spermatocytes in *Shoc1* KI homozygous (*Shoc1*^KI/KI^) and control mice (WT Hi-C dataset). (**D**) Normalized Hi-C interaction heatmaps (500 kb bins) for chromosome 2 in pachytene spermatocytes from adult *Shoc1* KI homozygous (*Shoc1*^KI/KI^) mice and controls (WT Hi-C dataset). (**E**) Pearson’s correlation heatmaps (500 kb bins) showing A/B compartmentalization patterns for chromosome 2 in pachytene spermatocytes from adult *Shoc1* KI homozygous (*Shoc1*^KI/KI^) mice and controls (WT Hi-C dataset), with first principal component eigenvalues (PCA1) displayed. (**F**) Normalized Hi-C interaction heatmaps (500 kb bins) for chromosome X in pachytene spermatocytes from adult *Shoc1* KI homozygous (*Shoc1*^KI/KI^) mice and controls (WT Hi-C dataset). (**G**) Pearson’s correlation heatmaps (500 kb bins) showing A/B compartmentalization patterns for chromosome X in pachytene spermatocytes from adult *Shoc1* KI homozygous (*Shoc1*^KI/KI^) mice and controls (WT Hi-C dataset), with PCA1 displayed. (**H**) Hi-C interaction matrix of chromosome 1 (50–55 Mb; 25 kb resolution) in pachytene spermatocytes from adult *Shoc1* KI homozygous (*Shoc1*^KI/KI^) mice and controls (WT Hi-C dataset). Triangles denote TADs. (**I**) TADs count in pachytene spermatocytes from adult *Shoc1* KI homozygous (*Shoc1*^KI/KI^) mice and controls (WT Hi-C dataset). (**J**) Average insulation scores ± 250 kb from TADs boundaries (25 kb resolution) in pachytene spermatocytes from adult *Shoc1* KI homozygous (*Shoc1*^KI/KI^) mice and controls (WT Hi-C dataset). Aggregated Hi-C interaction heatmaps flanking TAD boundaries (500 kb regions; **K**) or centered at CO-associated DSBs (2 Mb regions; **L**) in pachytene spermatocytes from adult *Shoc1* KI homozygous (*Shoc1*^KI/KI^) mice and controls (WT Hi-C dataset). Interactions normalized as observed/expected (Obs/Exp; 5 kb bins). (**M**) Box plots comparing Obs/Exp Hi-C interactions in 2 Mb regions centered at CO-associated DSBs in pachytene spermatocytes from adult *Shoc1* KI homozygous (*Shoc1*^KI/KI^) mice and controls (WT Hi-C dataset) using two-tailed Student’s *t*-test; **** *P* < .0001; error bars, mean ± SEM.

Chromatin undergoes dramatic 3D reorganization during meiosis (Fig. 5A), most remarkably exemplified by the loop-axis reconfiguration of pachytene chromosomes. However, direct evidence linking chromatin architecture defects to meiotic arrest remains elusive. Thus, we isolated pachytene spermatocytes from control and KI testes using a modified STA-PUT system (Supplementary Fig. S10A–C) and subsequently performed low-input Hi-C method (sisHi-C) to decipher chromatin architectural defects underlying meiotic arrest. The results of sisHi-C revealed significant alterations in the higher-order chromatin structure of *Shoc1* KI pachynema compared with that of the control group. Specifically, an overall decrease in distal interactions was observed in the mutant spermatocytes, as depicted in an interaction heatmap (Fig. 5B). This observation was further supported by P(s) curve analysis, which revealed a reduced interaction frequency in distal regions, indicating distinct principles of chromatin folding (Fig. 5C). In control pachynema, it was shown that distinct interchromosomal contact patterns were consistent with the alignment of chromosomes. Autosomal chromosomes in the control pachytene stage maintained a strong A/B compartment identity that was observed in the Hi-C contact map (Fig. 5D left panel) and Pearson correlation matrix (Fig. 5E left panel). Unlike the autosomal pattern, the X chromosome’s compartment structure was significantly lost in pachynema. Consistent with the idea that the clustering interactions are linked to transcription, we observed a near-complete loss of this clustering on the X chromosome as it became transcriptionally silenced in pachynema (Fig. 5F and G left panel). Previous data demonstrated that the pachytene chromatin organization is disrupted in *Sycp2*-mutant spermatocytes [42]. Similarly, we observed increased compartmentalization of both autosomes (Chromosome 2) and sex chromosomes (Chromosome X) in *Shoc1* KI pachynema (Fig. 5D–G right panel). In addition, the dynamics of TADs were also analyzed. Compared to control spermatocytes, *Shoc1* KI pachynema exhibited more refined and segmented TADs, with increased numbers and a subtle enhancement of boundary insulation (Fig. 5H–J).

During early meiotic prophase, DSBs preferentially emerged at chromatin loop anchorage sites along the chromosome axis, thereby facilitating homologous chromosome co-alignment. Notably, genomic regions with DSBs, particularly those linked with CO-designated DSBs, exhibited substantial reorganization of chromatin interaction networks concurrent with homologous pairing and axial alignment processes [46]. To investigate the spatiotemporal relationship between CO formation and chromatin architecture, we mapped CO hotspots in *Shoc1*-KI spermatocytes using established datasets combined with high-resolution sisHi-C interaction analysis. Pileup heatmap visualization revealed a significant reduction in chromatin interaction frequency flanking CO sites in the pachytene-stage nuclei of *Shoc1* KI mice compared with control mice (Fig. 5K–M). This attenuation of local chromatin connectivity near CO hotspots mirrored the dynamic chromatin reorganization patterns typically observed in zygotene spermatocytes [46, 50–52], suggesting that *Shoc1* mutation perturbs normal chromatin compaction dynamics during pachytene progression. These findings demonstrated that the variant within the XPF-like domain in *SHOC1* impaired dynamic 3D chromatin structure and CO region interactions.

### Shoc1 KI spermatocytes exhibited incomplete synapsis

**Figure 6.**
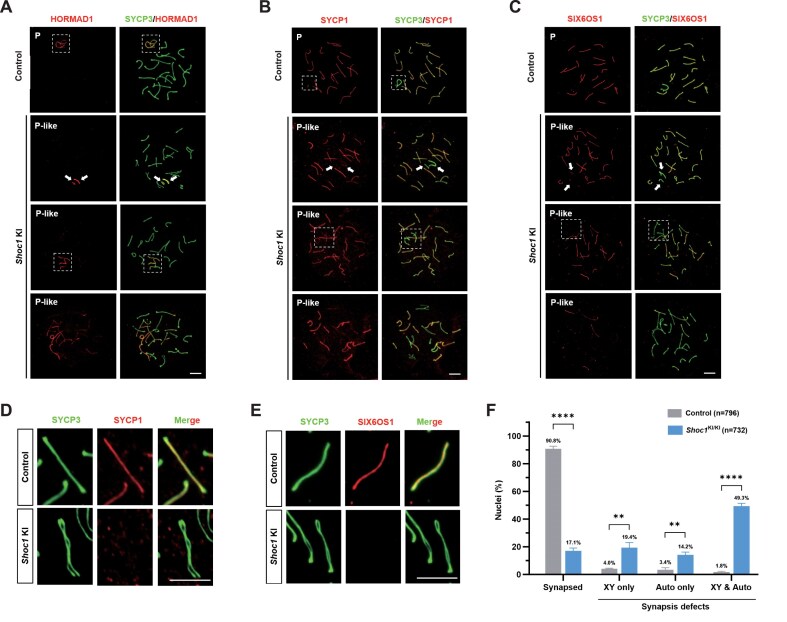
*Shoc1* KI spermatocytes exhibited incomplete synapsis. (A-C) Representative images of spread spermatocytes from adult *Shoc1* KI homozygous (*Shoc1*^KI/KI^) mice and littermate controls (*Shoc1*^KI/+^) co-stained with SYCP3 (green) and HORMAD1 (red, A), SYCP1 (red, B), SIX6OS1 (red, C). Scale bars, 10 μm. (D,E) Enlarged views of spread spermatocytes co-stained with SYCP3 (green) and SYCP1 (red, D), SIX6OS1 (red, E). Scale bars, 10 μm. (**F**) Quantification of synaptic defects in pachytene spermatocytes from adult *Shoc1* KI homozygous (*Shoc1*^KI/KI^) mice and littermate controls (*Shoc1*^KI/+^) using two-tailed Student’s t-test; **** *P* < 0.0001; ** *P* < 0.01; error bars, mean ± SEM; n, the total number of nuclei analyzed. XY, X and Y chromosomes; Auto, autosomes.

Since synapsis and HR are interdependent and closely coupled processes during meiotic prophase, we subsequently investigated the dynamics of chromosomal synapsis in *Shoc1* KI spermatocytes. We first stained the spermatocyte spreads with antibodies against SYCP3 and HORMAD1, which are localized along the unsynapsed chromosome axis in pachynema. In control mice, the HORMAD1 signal was observed only at unsynapsed sex chromosomal regions in the pachytene stage. However, in *Shoc1* KI spermatocytes, persistent HORMAD1 signals were detected on the autosomal chromosome axis (Fig. 6A). After further staining of the spermatocyte spreads for SYCP3 and SYCP1 (a transverse filament of the SC), as expected, the SYCP1 signals extended to the entire length of the autosomal axes and the PAR of the sex chromosomes in control pachytene spermatocytes. In *Shoc1* KI cells, the majority of pachytene-like cells with short and thick LEs displayed discontinuous and/or weak SYCP1 signals, suggesting that synaptic defects occurred after the mutation within XPF-like domain in *Shoc1* (Fig. 6B and D).

Furthermore, we stained the spermatocyte spreads with antibodies against SYCP3 and the CEs protein SIX6OS1 (Fig. 6C and E). Similar to the observation in SYCP1-staining spermatocytes, discontinuous SIX6OS1 signals were evident in the pachytene spermatocytes of *Shoc1* KI mice. Remarkably, in control mice, 90.8 ± 1.5% of pachytene spermatocytes achieved complete synapsis. However, in *Shoc1* KI mice, although 17.1 ± 1.6% of pachytene spermatocytes presented fully synapsed chromosomes, the remaining showed variable synaptic abnormalities. Among them, 14.2 ± 1.7% of cells presented synapsed sex chromosomes but with at least one pair of incompletely synapsed autosomes, 19.4 ± 3.0% of cells presented fully synapsed autosomes but with asynapsed sex chromosomes, and 49.3 ± 1.6% presented synapsis defects on both autosomal and sex chromosomes (Fig. 6F). Thus, these findings indicated that *Shoc1* mutation (p.Q646R) can lead to synaptic defects.

### The XPF-like domain in SHOC1 establishes a molecular barrier protecting autosome from meiotic silencing of unsynapsed chromatin

During meiosis, unsynapsed chromosomal regions are transcriptionally silenced through a conserved mechanism known as meiotic silencing of unsynapsed chromatin (MSUC). Specifically, the silencing of unsynapsed sex chromosomes is termed meiotic sex chromosome inactivation (MSCI) [53, 54]. Varying degrees of asynapsis could result in disturbance of the normal loading of MSUC proteins, impacting gene expression on autosomal and sex chromosomes and leading to extensive spermatocyte apoptosis [55, 56]. To investigate whether the MSUC is disrupted in *Shoc1* KI pachytene-like spermatocytes, RNA polymerase II (POL II) was used to detect transcriptional activity within chromosomes. In control pachynema, POL II signals were distinctly enriched on autosomes, consistent with transcriptional silencing via MSCI. In contrast, *Shoc1* KI cells exhibited a pronounced loss of POL II signals not only on sex chromosomes but also in adjacent autosomal regions (39.0 ± 1.7% in KI versus 3.1 ± 0.6% in control, *P* < .0001) (Supplementary Fig. S11A and B). Since the DNA damage response (DDR) pathway is essential for establishing and maintaining MSCI, we measured the expression of MDC1, a DDR factor critical for the amplification of DDR factors from the axis to the chromosome-wide domain [53], in *Shoc1* KI mice. Similarly, a greater percentage of trapped MDC1 signals was observed on both sexual chromosomes and autosomes in the KI group compared to the control group (53.0 ± 3.0% in the KI group versus 1.6 ± 0.5% in the control group, *P* < .0001) (Supplementary Fig. S11C and D). These results suggested that the overloaded MSUC in *Shoc1* KI pachytene-like spermatocytes can impede the expression of genes essential for meiotic progression, potentially contributing to the MMⅠ arrest.

**Figure 7.**
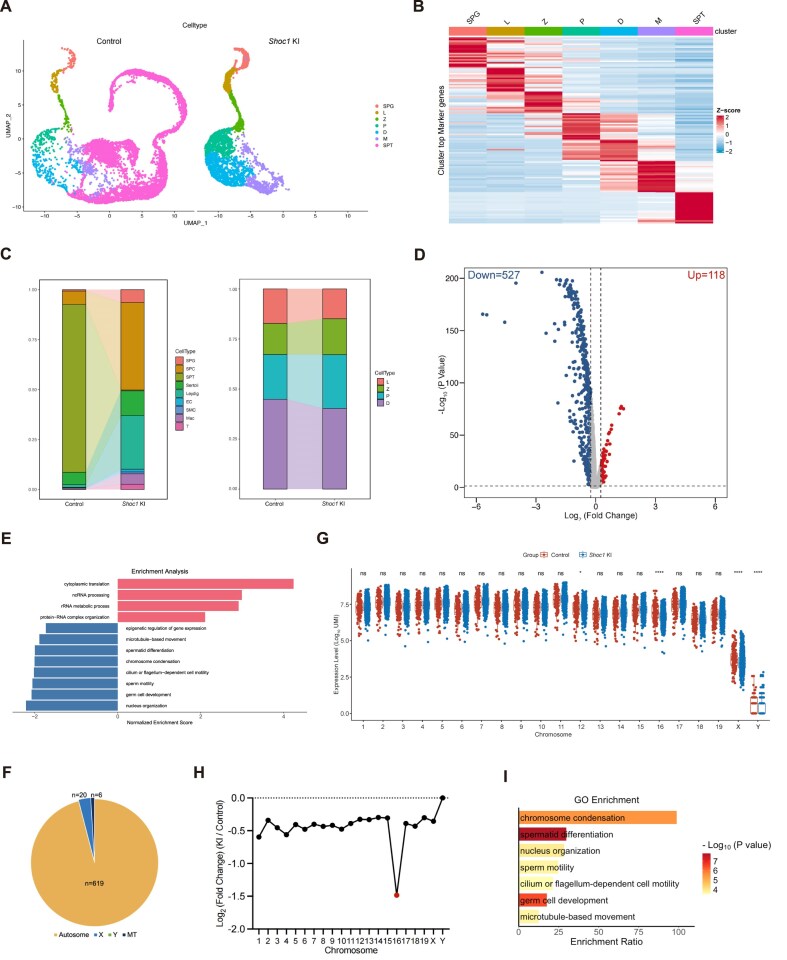
*Shoc1* KI led to overloaded MSUC and autosome-linked gene silencing in pachytene spermatocytes. (**A**) UMAP plots of testicular single-cell transcriptomes from adult *Shoc1* KI homozygous (*Shoc1*^KI/KI^) mice and littermate controls (*Shoc1*^KI/+^). (**B**) Heatmap displaying the top DEGs across germ cell subclusters. A gradient of blue to red indicates low to high expression levels. (**C**) Stacked bar charts quantifying cell type (left) and spermatocyte subtypes (right) proportions from adult *Shoc1* KI homozygous (*Shoc1*^KI/KI^) mice and littermate controls (*Shoc1*^KI/+^). SPG: spermatogonia; SPC: spermatocyte; SPT: spermatid; EC, endotheliocyte; SMC: smooth muscle cells; Mac: macrophages; T: peritubular myoid cells; L: leptotene spermatocyte; Z: zygotene spermatocyte; P: pachytene spermatocyte; D: diplotene spermatocyte. (**D**) Volcano plot of DEGs in pachytene spermatocytes from adult *Shoc1* KI homozygous (*Shoc1*^KI/KI^) mice and littermate controls (*Shoc1*^KI/+^); Cut-off: log_2_ (Fold Change) ≥ 0.25 or ≤ −0.25, *P* < .05. (**E**) GSEA of downregulated (blue) and upregulated (red) DEGs in pachytene spermatocytes from adult *Shoc1* KI homozygous (*Shoc1*^KI/KI^) mice and littermate controls (*Shoc1*^KI/+^). (**F**) Distribution of DEGs across autosomes, sex chromosome (**X**), and mitochondrial genome (MT). (**G**) UMI counts for genes on each chromosome across spermatogenesis stages using two-tailed Student’s *t*-test; *P < .05; ****P < .0001; ns, not significant; error bars, mean ± SEM. (**H**) Mean log_2_ (fold change) of autosomal and sex chromosomes gene expression. (**I**) GO analysis of downregulated DEGs on chromosome 16 in pachytene spermatocytes from adult *Shoc1* KI homozygous (*Shoc1*^KI/KI^) mice and littermate controls (*Shoc1*^KI/+^).

To confirm the association of the XPF-like domain in SHOC1 with MSUC and determine the consequences of MSUC overload in *Shoc1* KI mice, we compared the transcriptome of KI pachynema cells with that of control cells using 10 × Genomics scRNA-Seq. Distinct germ cell types were identified on the basis of uniform manifold approximation and projection (UMAP) and marker gene analysis from both control and *Shoc1*-KI testes (Fig. 7A and B). The cell proportions for each cell cluster and for spermatocytes at different stages are summarized in Fig. 7C. The findings revealed that the KI group lacked post-meiotic haploid spermatids, and the proportions of various substages of spermatocytes during meiosis were similar to our results from spread staining. Notably, the developmental trajectory of these pachytene-like spermatocytes in *Shoc1* KI mice did not differ significantly from that in control mice (Supplementary Fig. S12). Furthermore, with the cutoff of log_2_ (fold change) ≥ 0.25 or ≤ −0.25, a total of 645 DEGs, including 527 down-regulated genes and 118 up-regulated genes in *Shoc1* KI pachytene-like spermatocytes compared with WT, were identified (Fig. 7D). The results of GSEA of genes downregulated in *Shoc1*-KI spermatocytes compared with control pachytene spermatocytes included “nuclear organization,” “germ cell development,” “cilium or flagellum-dependent cell motility,” “chromosome condensation,” and “spermatid differentiation” (Fig. 7E). Given that the *Shoc1* KI pachytene spermatocytes exhibited overloaded MSUC, we compared mRNA levels between autosomes, X chromosome, and Y chromosome. We found that the majority of DEGs were distributed on the autosomes (619/645, 96.0%) rather than the sex chromosomes (X and Y chromosomes) (Fig. 7F). Intriguingly, genomic transcription mapping across chromosomes revealed that autosomal downregulated genes were predominantly clustered on chromosome 16 (Fig. 7G and H). Functional annotation demonstrated a strong association of these chromosome 16 genes with critical reproductive processes, including “chromosome condensation,” “spermatid differentiation,” and “germ cell development” through rigorous GO enrichment analyses, mechanistically linking this dysregulation to apoptosis during MMI and meiotic arrest (Fig. 7I). Overall, our findings revealed that the XPF-like domain in SHOC1 establishes a molecular barrier preventing autosome intrusion into the sex body compartment, thereby protecting critical autosomal loci from MSUC. Additionally, chromosome 16 exhibits heightened MSUC vulnerability, as shown by its disproportionate transcriptional suppression in *Shoc1* mutation within XPF-like domain.

## Discussion

As the basis of sexual reproduction, meiosis is required to ensure genome stability and heritable diversity through the generation of haploid gametes and the formation of DSBs, homologous pairing, synapsis, HR, and CO formation. Defects in evolutionary molecular machines can result in meiotic arrest or aneuploidy, ultimately damaging human fertility. Our findings revealed the critical role of the XPF-like domain in SHOC1 as a molecular scaffold regulating CO formation and synapsis during meiosis. Species-specific interactome analyses identified distinct SHOC1 complexes between humans and mice *in vitro*. The p.Q590R variant within the XPF-like domain in *SHOC1* impaired DSBs repair by compromising SHOC1’s ability to bind branched DNA structures and the recruitment of M1AP, REDIC1, as well as ZMM factors to recombination intermediates, ultimately abolishing CO formation and causing meiotic arrest. Notably, Hi-C analyses revealed that mutant pachytene spermatocytes exhibit disrupted chromatin architecture–increased TAD insulation, elevated compartmentalization, and weakened interactions at CO sites–phenocopying zygotene-like chromatin states. We further identified the novel role of SHOC1 in protecting autosomes from the XY body, with transcriptomic data showing chromosome 16-specific silencing of meiotic regulators through overloaded MSUC activation.

In the conserved Zip2-Zip4-Spo16 (ZZS) trimeric complex of yeast, Zip4 interacts with the N-terminal domain of Zip2, whereas Spo16 binds specifically to its C-terminal XPF-like domain, which is required for the formation of class Ⅰ interfering COs and polymerization of the SC [31, 32]. Recent studies reported two novel proteins M1AP and REDIC1 in mouse testes can interact and cooperate with the ZZS complex to stabilize recombination intermediates [22, 23]. To further confirm the interaction between SHOC1 and these two proteins and other ZZS components, we performed co-IP in lysates of HEK293T cells co-expressing human or mSHOC1 with TEX11, SPO16, M1AP, or REDIC1. Comparative analyses revealed pronounced evolutionary divergence in the molecular organization of the SHOC1 complex. In the mouse system, co-IP and Bi-FC data revealed pairwise interactions among SHOC1, TEX11, and SPO16, suggesting that this complex functions as a highly stable, pre-assembled functional module. In contrast, the human SHOC1 complex exhibits distinct interaction dynamics. *In vitro* co-IP and Bi-FC assays revealed an interaction exclusively between SHOC1 and TEX11. However, due to the lack of commercially available antibodies, we were unable to confirm the interaction between human SHOC1 and C1orf146 by co-IP *in vivo*. This interaction could be further investigated in the future by generating IP-compatible antibodies against SHOC1 and C1orf146. Furthermore, we observed that interactions with human M1AP/REDIC1 are not affected by various SHOC1 truncations (ΔF1, ΔF2, ΔF3, and ΔF4), which, coupled with our findings, raise the possibility that SHOC1 associates with M1AP/REDIC1 indirectly or via multivalent interactions. Similarly, the interactions between mouse TEX11 and SHOC1 were not disrupted by various SHOC1 truncations (ΔF1, ΔF2, ΔF3, and ΔF4), however, C-terminal region of SHOC1 was not required for the binding. Notably, SHOC1 truncations (ΔF1 and ΔF2) retained the ability to interact with TEX11, suggesting multivalent interactions between mSHOC1 and mTEX11, similar with the interactions between CE components (specifically SYCE1 and SIX6OS1) as previously reported [40]. Taken together, these findings highlight a previously unrecognized species-specific interactome within the SHOC1 complex, thereby refining the understanding of its functional role in human meiosis.

Several studies have reported the clinical relevance of *SHOC1* variants in varying severity of meiotic arrest phenotypes. Wang *et al*. reported their findings of bi-allelic *SHOC1* variants in three NOA-affected patients, of which two were homozygous for the same LoF variant (c.231_232del:p.L78Sfs*9), and one is heterozygous for two different missense variants (c.1978G > A:p.A660T; c.4274G > A:p.R1425H). Testicular biopsy of one patient revealed impairment of spermatocyte maturation [30]. In contrast, Csilla *et al*. reported that 4.2% of spermatocytes in a patient homozygous for the frameshift variant c.797delT (p.L266Qfs*6) achieved XY body formation and progressed to MMI [57]. Here, we expanded the genetic landscape of meiotic arrest by screening for pathogenic *SHOC1* variants and revealed severe zygotene arrest without XY body formation in the testicular sections of probands. These abnormalities were similar to those in germ-cell specific (conditional knockout) cKO and systemic KO mouse models, which were arrested at the zygotene-like stage [14, 30]. For several of the identified variants, we demonstrated functional data linking the impaired integrity of encoded proteins due to different frameshift types or aberrant mRNA splicing to downstream cellular effects. Importantly, our results provided strong evidence for the clinical relevance and emphasized the necessity for *SHOC1* gene screening in NOA diagnosis.

Given the high evolutionary conservation of the XPF-like domain in SHOC1 across species, investigations of the critical residues and systematic characterization of molecular mechanisms are imperative to elucidate for elucidating the essential role of the XPF-like domain during meiotic progression. While previous investigations have primarily utilized bioinformatic analyses and *in vitro* biochemical approaches [17, 31], direct *in vivo* evidence remains limited. In this study, we identified a missense variant within the XPF-like domain (p.Q590R) through genetic screening and established a CRISPR/Cas9-edited KI mouse model, which exhibited not only CO elimination but also synaptic defects. Recent work on a complete *Shoc1*-null mouse model revealed even more severe phenotypes, showing complete arrest at a zygotene-like stage with defective synapsis, unresolved DSBs, and absence of both ZMM protein-associated recombination intermediates and MLH1-marked class Ⅰ COs [14, 16]. In comparison, our KI model and the reported *Shoc1*^hyp/hyp^ mutant (expressing reduced levels of truncated SHOC1) (17)both displayed MMI arrest with univalent chromosomes. This phenotype closely resembles those observed in TEX11 [58], M1AP [22], and REDIC1 [23] deficient mouse models, collectively suggesting that the functional role of the Q646 residue (corresponding to human Q590 residue) within the XPF-like domain in SHOC1 aligns with the critical transition from early recombination intermediates to mature COs during late prophase I.

In the current study, unlike the early loss in *Shoc1* KO mice, the population of prophase I spermatocytes in our *Shoc1* KI model remained stable. This effect may result from the activation of distinct meiotic checkpoints, including the DNA damage checkpoint, the synapsis checkpoint and the SAC. The former two are also referred to as the pachytene checkpoints, as they guarantee that synapsis and recombination of germ cells occur normally. The SAC is responsible for accurate segregation of homologous chromosomes [59, 60]. In our study, p-CHK1 and BRCA1 (the mediators of pachytene checkpoint) levels outside the sex body did not significantly increase at the pachytene-like stage in *Shoc1* KI spermatocytes. However, the SAC was highly activated, as indicated by the significantly increased number of BUB3-positive spermatocytes. Nevertheless, in a subset of spermatocytes, BRCA1-positive autosomal signals were detected within the sex body. These results suggested that spermatocytes with partial synapsis failure associated with autosome asynapsis in sex body, and are able to progress beyond the diplotene stage and subsequently undergo cell death at metaphase through SAC activation.

The more severe synapsis defects observed in our *Shoc1* KI mice than in these other models underscored the central role of SHOC1 complex as a molecular scaffold in regulation of both synapsis and CO formation during meiosis. It has been reported that mutations in multiple DNA-binding surfaces on Zip2-Spo16 severely compromise DNA binding, supporting a model in which the XPF-like domain cooperates to bind with branched DNA structures [31]. Thus, we investigated the effects resulting from the current mutation within the XPF-like domain on its ability to bind branched DNA structures using molecular docking. The findings demonstrated that the substitution-induced conformational changes appeared to mechanistically impair branched DNA (D-loop) binding ability of the XPF-like domain in both human and mSHOC1.

Chromatin undergoes drastic reprogramming during meiosis [46, 50–52]. Previous studies have examined dynamics of the 3D meiotic chromatin architecture in mice and rhesus monkeys, in which chromosomal TADs undergo dissolution and re-establishment during spermatogenesis [42, 51]. This is accompanied by the emergence of a unique chromatin configuration as the SC forms, characterized by highly refined, transcriptionally correlated chromatin compartments. Notably, the fact that both SYCP2 and TOP6BL deficient mice, in which the SC failed to be established, exhibited the restoration of TADs and increased conventional compartmentalization in spermatocytes, suggesting that the SC may restrict TADs and promote local compartments [42]. In the present study, sisHi-C analyses revealed that *Shoc1* KI disrupts hierarchical chromatin organization, which is concomitant with meiotic arrest. While compartment/TAD features persist in *Shoc1* mutant pachynema, diminished distal interactions and failed chromosome alignment revealed the essential function of SHOC1 in coordinating higher-order chromatin topology. Moreover, the attenuation of chromatin connectivity at CO hotspots in KI group suggested that SHOC1 can protect local chromatin compaction during pachytene, ensuring proper coordination between SC assembly and homology-directed DSBs repair.

Intriguingly, our investigation revealed that MSUC was overloaded in the pachytene spermatocytes of *Shoc1*-KI mice. scRNA sequencing analysis revealed that the KI group exhibited autosomal transcriptional suppression, especially in chromosome 16. Similarly, in spermatocytes carrying the T (X;16)16H reciprocal X–autosome translocation, varying degrees of asynapsis were observed, accompanied by extensive spermatocyte loss due to apoptosis. Notably, the unsynapsed chromosome 16 segment was always Cot-1 (indicative of nascent transcripts)–negative and γH2AX-positive, suggesting unsynapsed regions of chromosome 16 undergo transcriptional silencing via MSUC [61]. These findings indicated that chromosome 16 can be transcriptionally repressed through MSUC.

In the current study, chromosome 16 displayed the most significant differential expression in pachytene spermatocytes of *Shoc1* KI mice. It was revealed that the downregulated genes on chromosome 16 largely fall into two functional categories that may underlie male infertility. First, genes directly involved in meiosis, such as *Sod1*, maintain genomic integrity in spermatocytes by preserving redox homeostasis and facilitating DNA damage repair, which is essential for homologous recombination [62]. Second, transcripts influencing post-meiotic spermatid differentiation, such as *Prm1*/*2/3, Tnp2*, and *Tssk1*, are transcribed during meiosis but translated later during spermiogenesis. *Prm1*/*2/3* and *Tnp2* orchestrate histone-protamine transition in spermatids [63–65], whereas Tssk1 regulates chromatoid body-mediated cytodifferentiation [66]. Critically, owing to this unique transcription–translation uncoupling characteristic of spermatogenesis, the depletion of these stored mRNAs in meiotic prophase, despite their late protein functions, may indirectly disrupt meiosis progression by altering RNA-binding protein sequestration or impairing ribonucleoprotein granule dynamics, which are crucial for the temporal regulation of meiotic transcripts. Notably, chromosome 16 predisposition is clinically relevant, as trisomy 16 represents a frequent autosomal anomaly in early gestational loss [67]. While these data suggested chromosomal specificity in transcriptional perturbation, the precise mechanisms underlying how the XPF-like domain mutation in *SHOC1* mediates autosomal MSUC overload remain to be fully elucidated.

This study has several limitations that should be acknowledged. Molecular docking, an *in silico* assay, may introduce certain biases compared with *in vitro* and *in vivo* DNA binding assays. Moreover, expression and purification of recombinant SHOC1 was technically challenging, preventing us from conducting *in vitro* DNA-binding assays in the current study. Future investigations could address this limitation by resolving the *in situ* structure of the SHOC1–DNA complex to further assess how the variant affects the interaction between SHOC1 and branched recombination intermediates.

In conclusion, our findings revealed species-specific differences between human and mSHOC1 complex, and revealed the critical role of the XPF-like domain in SHOC1 as a molecular scaffold that regulates synapsis and CO formation during meiosis. The disorder of the XPF-like domain in SHOC1 impaired DSB repair by compromising its ability to bind branched DNA structures and the recruitment of M1AP, REDIC1, and ZMM factors to recombination intermediates, ultimately abolishing CO formation. Furthermore, it disrupted dynamic 3D chromatin structure in pachytene spermatocytes and induced defects in homologous chromosome synapsis. Importantly, the XPF-like domain in SHOC1 was revealed to prevent autosome intrusion into the sex body compartment, thereby protecting critical autosomal loci from MSUC.

## Supplementary Material

gkag558_Supplemental_File

## Data Availability

The scRNA-seq matrix data and the Hi-C data generated in this study have been deposited in Dryad: https://doi.org/10.5061/dryad.3n5tb2rvx Control Hi-C dataset from mouse cells at the pachytene stage was obtained from published work (accession no. GSE109344) [42]. All other relevant data supporting the key findings of this study are available within the article and its supplementary information files or from the corresponding authors upon reasonable request. Source data are provided with this paper.
