# Supplementary material for: XPF-like domain in human SHOC1 is required for crossover formation and protecting autosome from MSUC
Source: Nucleic Acids Res. 2026 Jun 8;54(11):gkag558. doi: 10.1093/nar/gkag558 (PMC13245411; doi:10.1093/nar/gkag558)
Supplement: gkag558_Supplemental_File [file gkag558_supplemental_file.pdf]

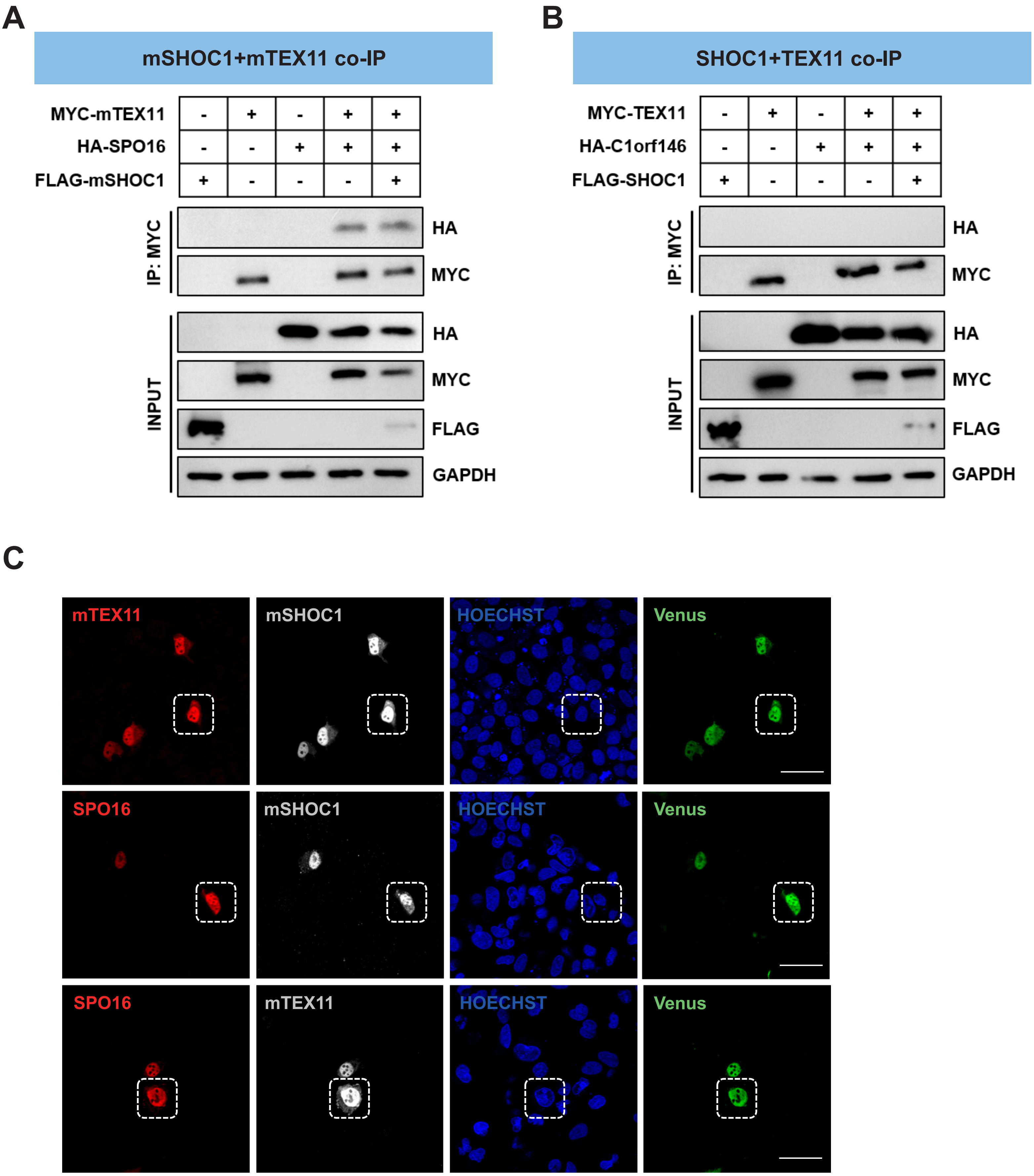
A
mSHOC1+mTEX11 co-IP
MYC-mTEX11
HA-SPO16
FLAG-mSHOC1
IP: MYC
HA
MYC
INPUT
HA
MYC
FLAG
GAPDH
B
SHOC1+TEX11 co-IP
MYC-TEX11
HA-C1orf146
FLAG-SHOC1
IP: MYC
HA
MYC
INPUT
HA
MYC
FLAG
GAPDH
C
mTEX11
mSHOC1
HOECHST
Venus
SPO16
mSHOC1
HOECHST
Venus
SPO16
mTEX11
HOECHST
Venus

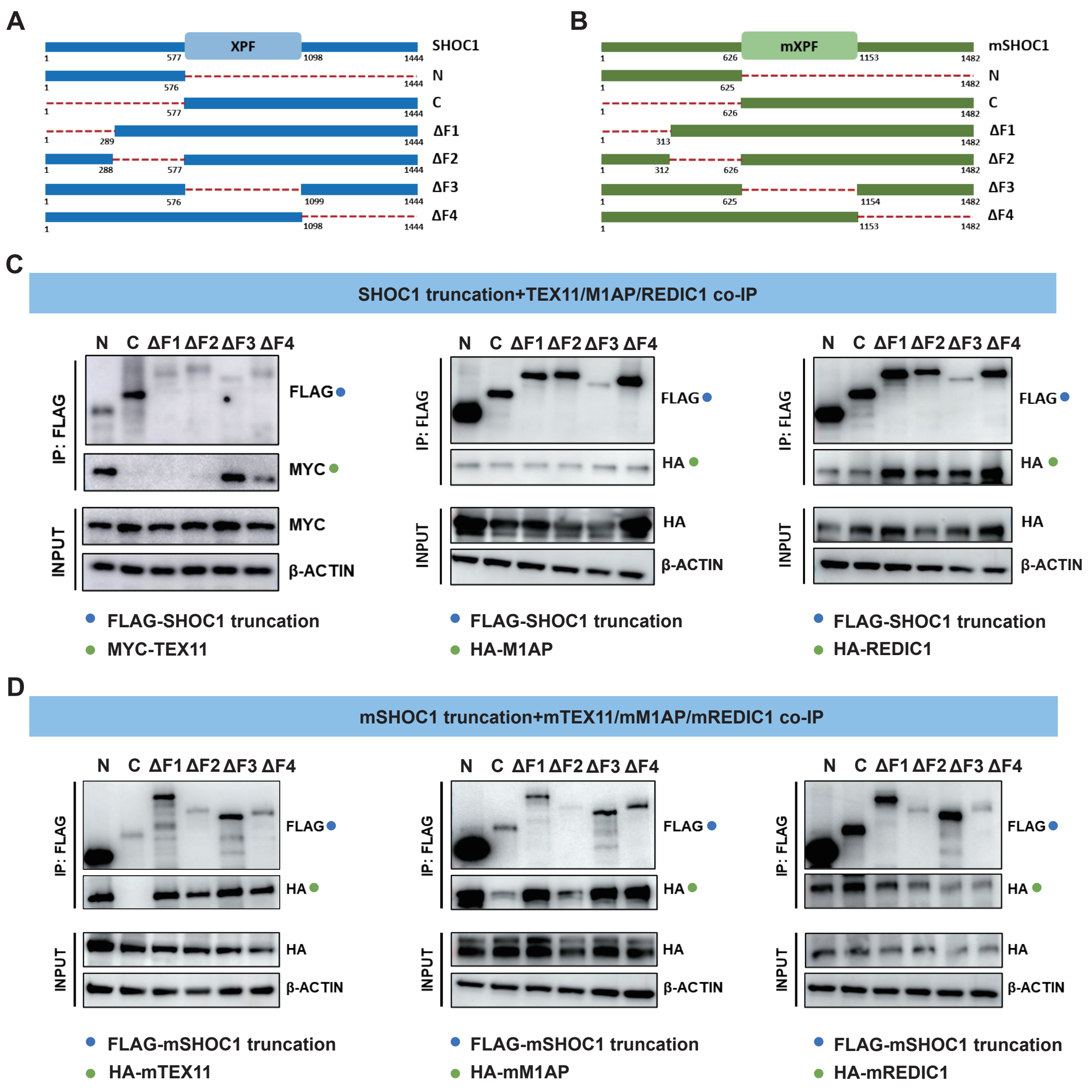
A
XPF
SHOC1
N
C
ΔF1
ΔF2
ΔF3
ΔF4
B
mXPF
mSHOC1
C
SHOC1 truncation+TEX11/M1AP/REDIC1 co-IP
IP: FLAG
INPUT
FLAG
MYC
HA
β-ACTIN
FLAG-SHOC1 truncation
MYC-TEX11
HA-M1AP
HA-REDIC1
D
mSHOC1 truncation+mTEX11/mM1AP/mREDIC1 co-IP
FLAG-mSHOC1 truncation
HA-mTEX11
HA-mM1AP
HA-mREDIC1

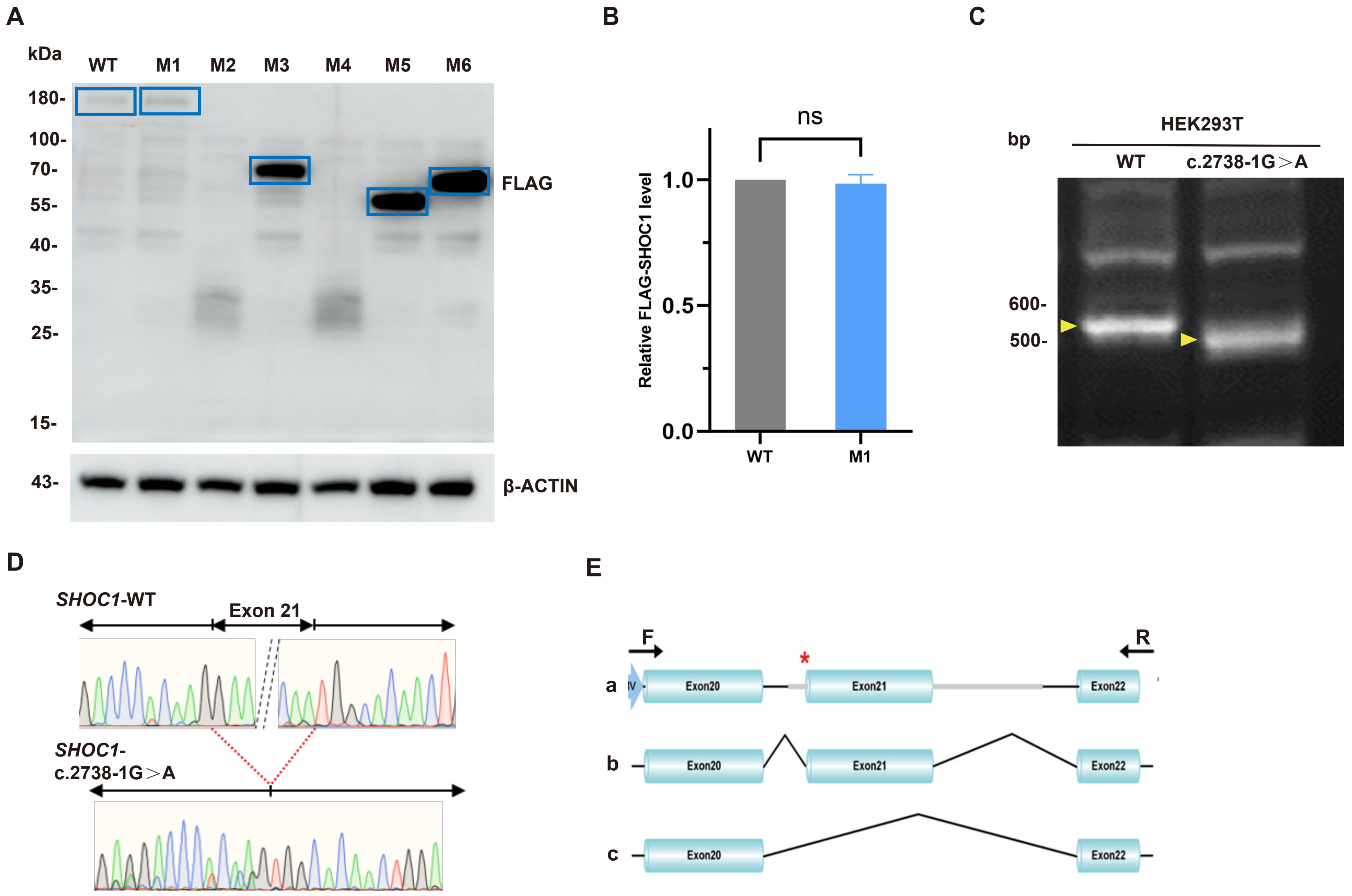

A
kDa
WT
M1
M2
M3
M4
M5
M6
180-
100-
70-
55-
40-
35-
25-
15-
FLAG
43-
β-ACTIN
B
ns
Relative FLAG-SHOC1 level
1.0
0.5
0.0
WT
M1
C
HEK293T
bp
WT
c.2738-1G＞A
600-
500-
D
SHOC1-WT
Exon 21
SHOC1-c.2738-1G＞A
E
F
R
a
b
c
Exon20
Exon21
Exon22

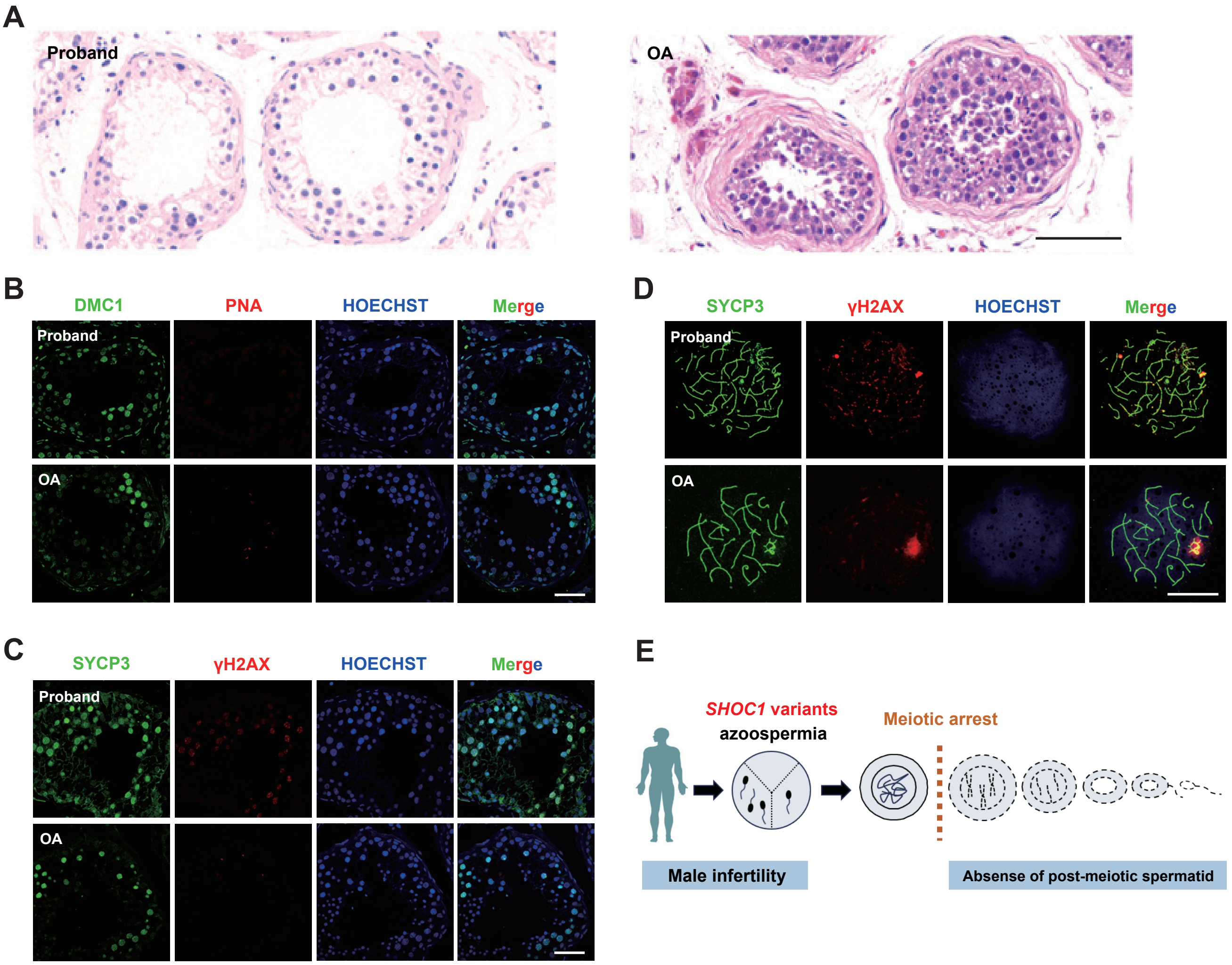
A
Proband
OA
B
DMC1
PNA
HOECHST
Merge
Proband
OA
C
SYCP3
γH2AX
HOECHST
Merge
Proband
OA
D
SYCP3
γH2AX
HOECHST
Merge
Proband
OA
E
SHOC1 variants
azoospermia
Meiotic arrest
Male infertility
Absense of post-meiotic spermatid

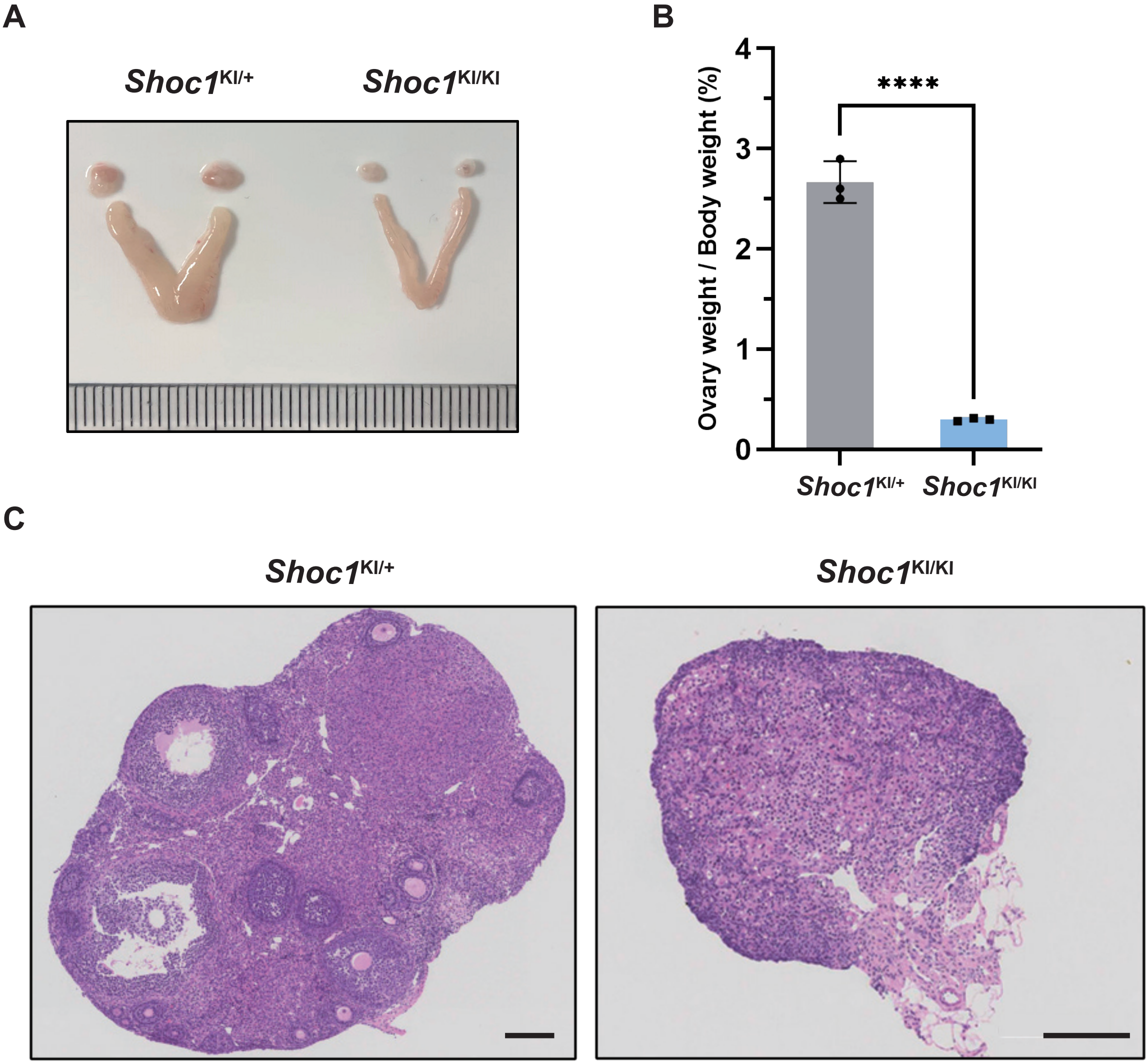
A
Shoc1KI/+
Shoc1KI/KI
B
Ovary weight / Body weight (%)
0
1
2
3
4
****
Shoc1KI/+
Shoc1KI/KI
C
Shoc1KI/+
Shoc1KI/KI

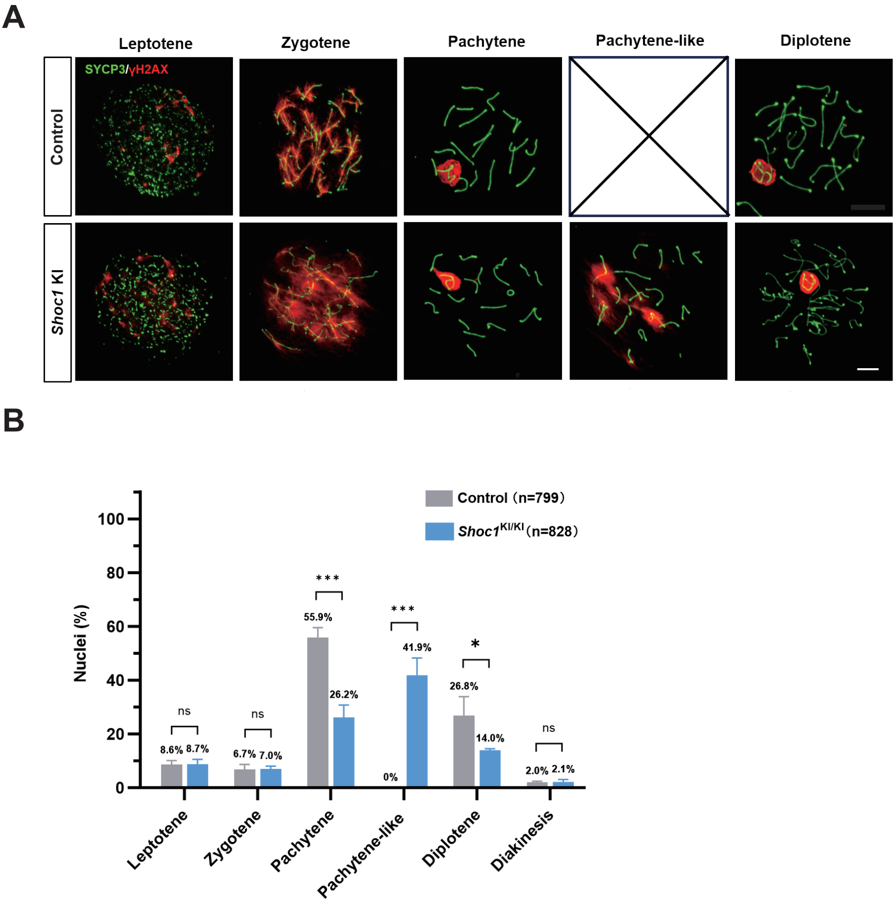
A
Leptotene
Zygotene
Pachytene
Pachytene-like
Diplotene
Control
Shoc1 KI
SYCP3/γH2AX
B
Control（n=799）
Shoc1KI/KI（n=828）
Nuclei (%)
100
80
60
40
20
0
ns
ns
***
***
*
ns
8.6%
8.7%
6.7%
7.0%
55.9%
26.2%
0%
41.9%
26.8%
14.0%
2.0%
2.1%
Leptotene
Zygotene
Pachytene
Pachytene-like
Diplotene
Diakinesis

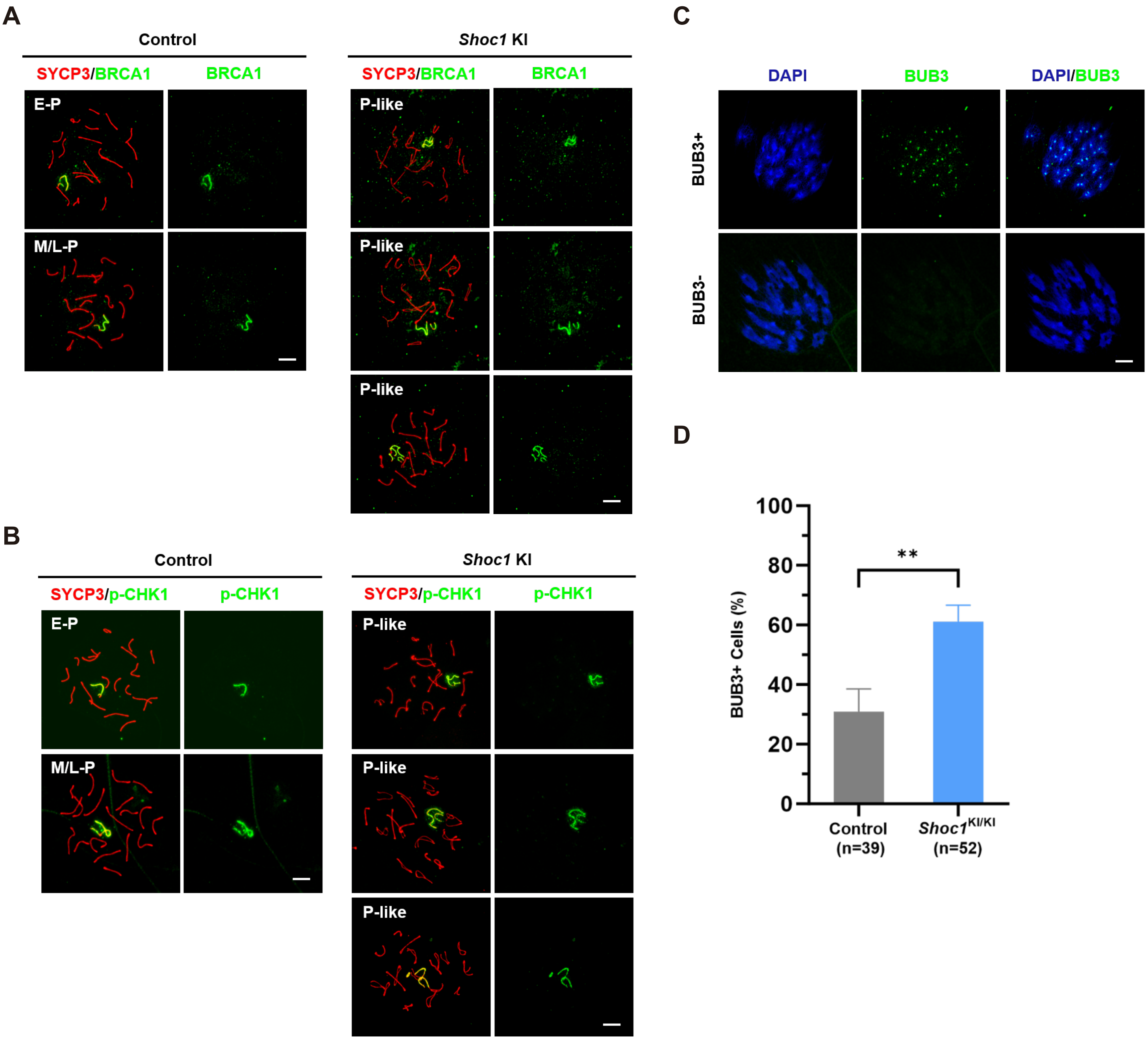
A
Control
SYCP3/BRCA1
BRCA1
E-P
M/L-P
Shoc1 KI
SYCP3/BRCA1
BRCA1
P-like
P-like
P-like
B
Control
SYCP3/p-CHK1
p-CHK1
E-P
M/L-P
Shoc1 KI
SYCP3/p-CHK1
p-CHK1
P-like
P-like
P-like
C
DAPI
BUB3
DAPI/BUB3
BUB3+
BUB3-
D
BUB3+ Cells (%)
100
80
60
40
20
0
**
Control
(n=39)
Shoc1KI/KI
(n=52)

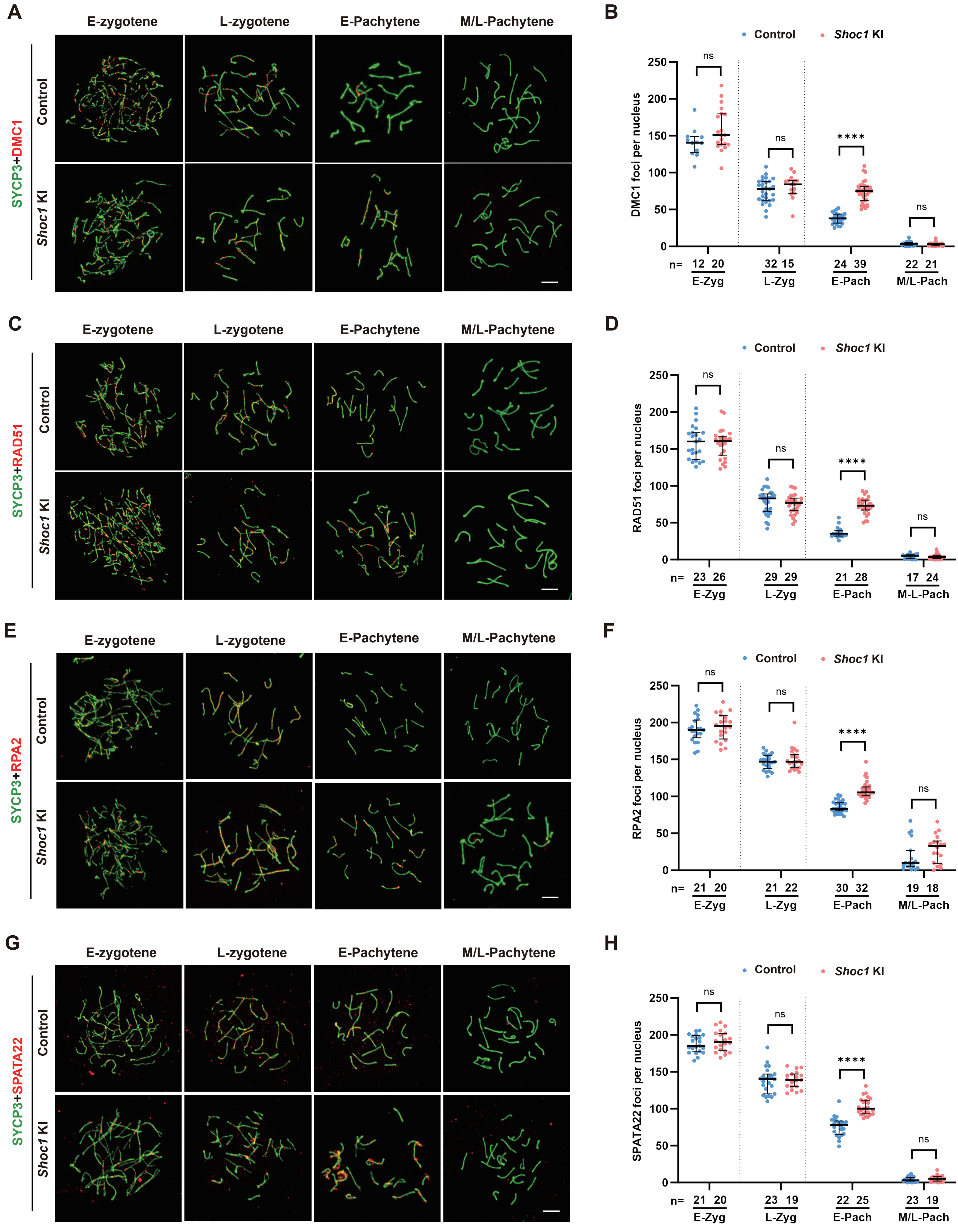

A
E-zygotene
L-zygotene
E-Pachytene
M/L-Pachytene
SYCP3+DMC1
Control
Shoc1 KI
B
Control
Shoc1 KI
DMC1 foci per nucleus
ns
ns
****
ns
n= 12 20 32 15 24 39 22 21
E-Zyg
L-Zyg
E-Pach
M/L-Pach
C
E-zygotene
L-zygotene
E-Pachytene
M/L-Pachytene
SYCP3+RAD51
Control
Shoc1 KI
D
Control
Shoc1 KI
RAD51 foci per nucleus
ns
ns
****
ns
n= 23 26 29 29 21 28 17 24
E-Zyg
L-Zyg
E-Pach
M-L-Pach
E
E-zygotene
L-zygotene
E-Pachytene
M/L-Pachytene
SYCP3+RPA2
Control
Shoc1 KI
F
Control
Shoc1 KI
RPA2 foci per nucleus
ns
ns
****
ns
n= 21 20 21 22 30 32 19 18
E-Zyg
L-Zyg
E-Pach
M/L-Pach
G
E-zygotene
L-zygotene
E-Pachytene
M/L-Pachytene
SYCP3+SPATA22
Control
Shoc1 KI
H
Control
Shoc1 KI
SPATA22 foci per nucleus
ns
ns
****
ns
n= 21 20 23 19 22 25 23 19
E-Zyg
L-Zyg
E-Pach
M/L-Pach

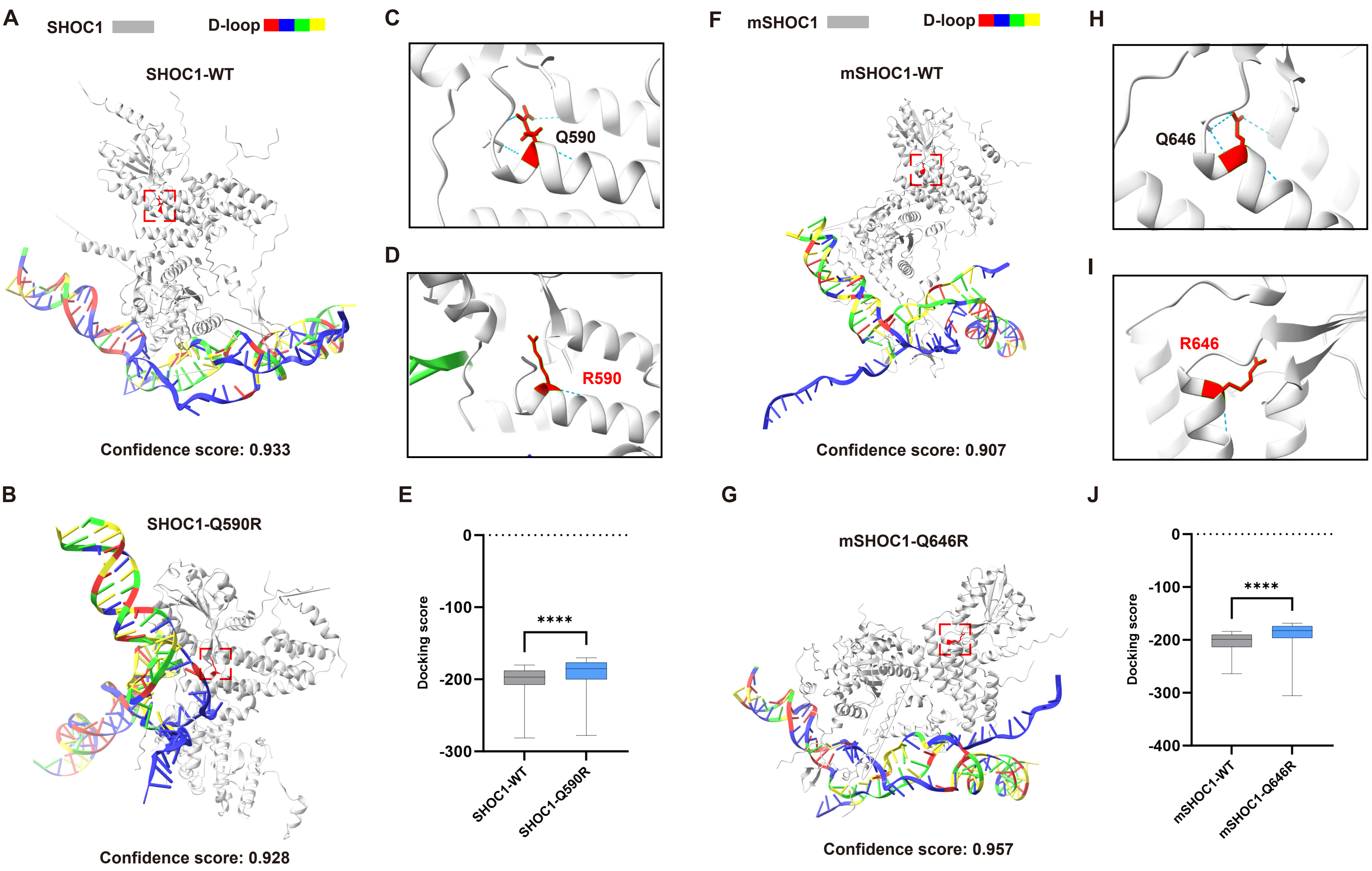
A
SHOC1
D-loop
SHOC1-WT
Confidence score: 0.933
B
SHOC1-Q590R
Confidence score: 0.928
C
Q590
D
R590
E
Docking score
0
-100
-200
-300
****
SHOC1-WT
SHOC1-Q590R
F
mSHOC1
D-loop
mSHOC1-WT
Confidence score: 0.907
G
mSHOC1-Q646R
Confidence score: 0.957
H
Q646
I
R646
J
Docking score
0
-100
-200
-300
-400
****
mSHOC1-WT
mSHOC1-Q646R

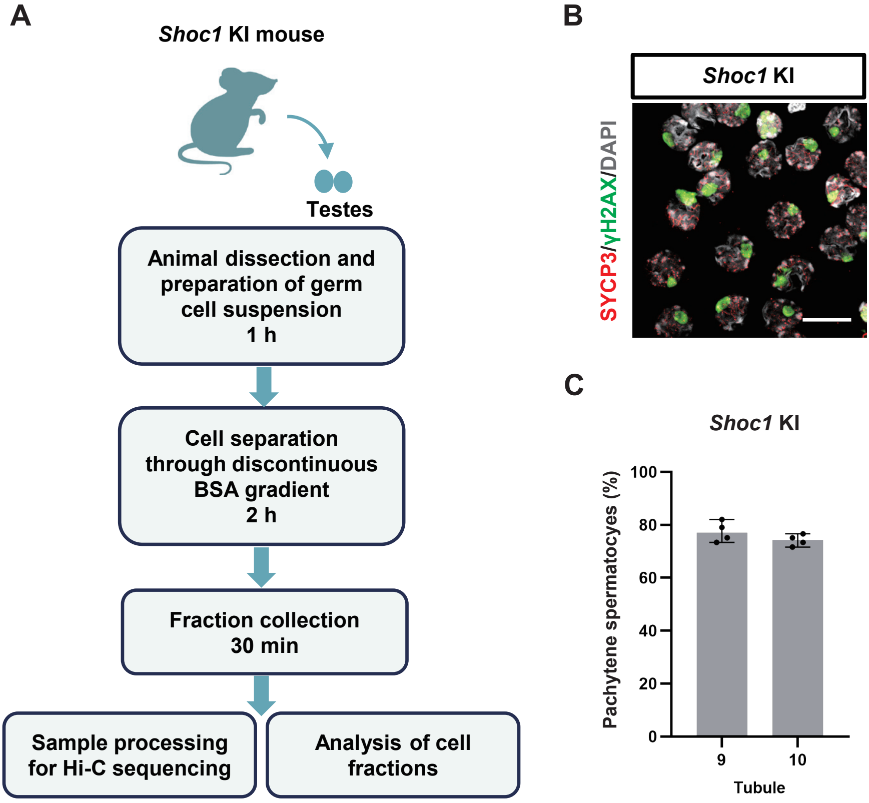
A
Shoc1 KI mouse
Testes
Animal dissection and preparation of germ cell suspension 1 h
Cell separation through discontinuous BSA gradient 2 h
Fraction collection 30 min
Sample processing for Hi-C sequencing
Analysis of cell fractions
B
Shoc1 KI
SYCP3/γH2AX/DAPI
C
Shoc1 KI
Pachytene spermatocyes (%)
0
20
40
60
80
100
9
10
Tubule

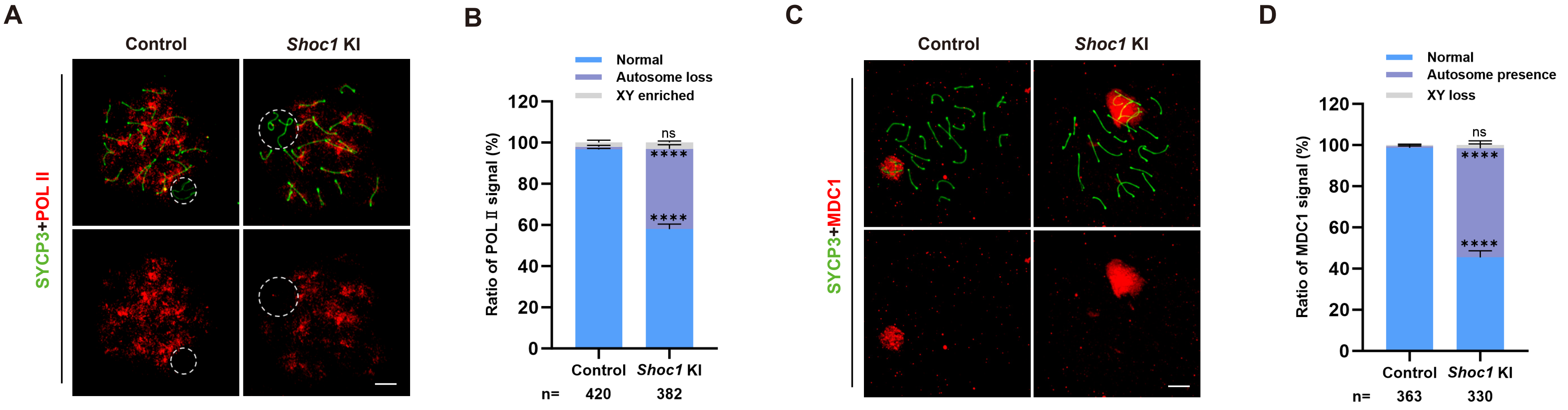
A
Control
Shoc1 KI
SYCP3+POL II
B
Normal
Autosome loss
XY enriched
Ratio of POL II signal (%)
0
20
40
60
80
100
120
ns
****
****
Control
Shoc1 KI
n=
420
382
C
Control
Shoc1 KI
SYCP3+MDC1
D
Normal
Autosome presence
XY loss
Ratio of MDC1 signal (%)
0
20
40
60
80
100
120
ns
****
****
Control
Shoc1 KI
n=
363
330

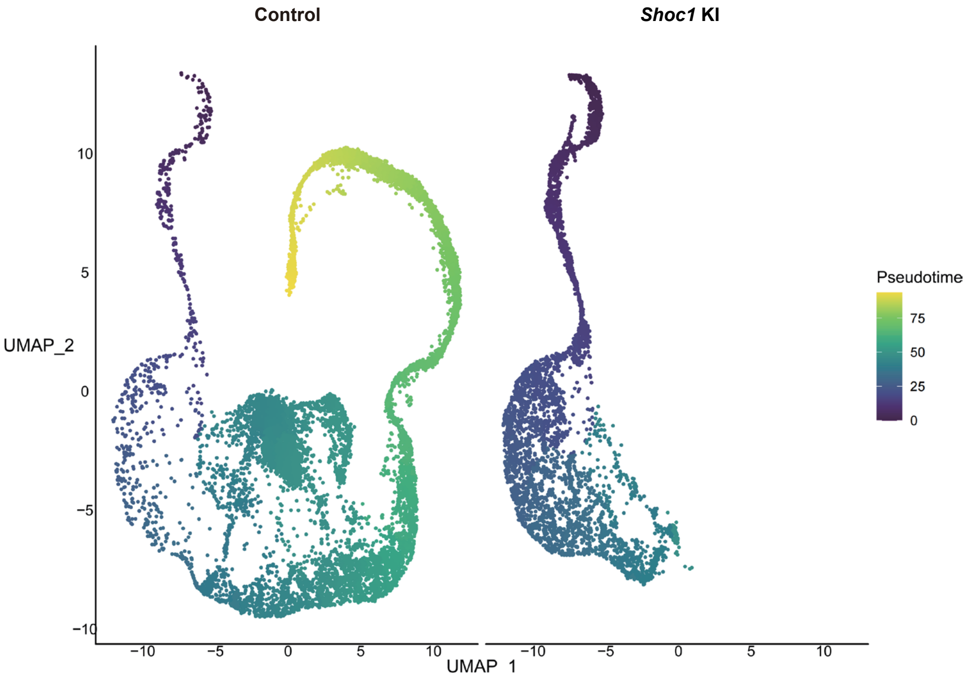

Control
Shoc1 KI
UMAP_2
10
5
0
−5
−10
−10
−5
0
5
10
−10
−5
0
5
10
UMAP_1
Pseudotime
75
50
25
0

Supplementary Table S1. Information of the primers.

Information	Name	Sequence (5'-3')
Primers for human gDNA amplification and Sanger sequencing (5’-3’)	M1: c.A1769G-F	AGGCAGGAGAATAGCTTGAATC
	M1: c.A1769G-R	GGCGAACAGCATCACTTACTA
	M2: c.416_419del-F	CTGCACCCGGCCAATAA
	M2: c.416_419del-R	ATACAGCCAAGGCTGAGAAC
	M3: c.C1582T-F	GACCAAGAACCAGTAAACAGAATAA
	M3: c.C1582T-R	GAGACAGGACTGACAGCTAAA
	M4: c.231_232del-F	CTTCTCTGTGTCTGTTGGAATTTG
	M4: c.231_232del-R	CTTAGAGTTATGCAGGTGGATCTT
	M5: c.1194delA-F	CCAGTGTGGTTCTGTTTATTGTG
	M5: c.1194delA-R	ATGGGACCTAGAGAAATCCAAAG
	M6: c.1347delT-F	CCAGTGTGGTTCTGTTTATTGTG
	M6: c.1347delT-R	AGTCTTTGAGGTGCAAGTCTTAT
	M7: c.G2738-1A-F	ACATTTCAGGTAGAGAATGACCC
	M7: c.G2738-1A-R	ACATTTCAGGTAGAGAATGACCC
Primers for genotyping of *Shoc1* KI mice (5’-3’)	F	GTTATCAGTGGTCACTCCATCCC
	R	AGATTCACTGAGTATCTTCTGGGCT
sgRNA for constructing *Shoc1* KI mice (5’-3’)		GAGACAGTATGCTTGGCACTGGG
Donor oligo for constructing *Shoc1* KI mice (5’-3’)		GACACAGCACACTACCCTTTTCACACACACTGATTTCAGTGCTGTTATTTTTCAGACACCCGGTGCCAAGCATACTGTCTCCTAGAAGCCACAGCGAATCCTATCTTAAAGGAGCTTGTTTGC
Primers for minigene of gDNA flanking the *SHOC1* M7 variant (5’-3’)	F	ATGGATGTTTTCCCAGTCACAGGTATTTATTCTTTTCTTATTGATCTGTAA
	R	GGATATCAGCTGGATGGCAATACTCATTCTCAATATTATCAATGACTCAA
Primers for constructing plasmids for Co-IP (5’-3’)	pCMV-SHOC1-FLAG-F	CTTGCGGCCGGCCGCGAATTCATGACAGATACCTCAGTCTTGGACC
	pCMV-SHOC1-FLAG-R	CCTCTAGAGTCGACTGGTACCTCAAAAAAACCTCAGCCGAGTC
	pcDNA-TEX11-MYC-F	GATTACGCCAAGCTTGGTACCATGGACAATGATGATTTTTTTCCATGGACT
	pcDNA-TEX11-MYC-R	TGCTGGATATCTGCAGAATTCCTAATCTGACTTGCTCCAGTAGCCA
	pcDNA-C1orf146-MYC-F	GAGGATCTGAAGCTTGGTACCATGGCTGA

	AAGTGGAAAAGAAAA
pcDNA-C1orf146-MYC-R	TGCTGGATATCTGCAGAATTCCTAATTTGG GTTAACTGAATCACTATTCA
pcDNA-C1orf146-HA-F	CTTGGTACCGAGCTCGGATCCATGGCTGA AAGTGGAAAAGAAAA
pcDNA-C1orf146-HA-R	TGCTGGATATCTGCAGAATTCCTAATTTGG GTTAACTGAATCACTATTCA
pcDNA-M1AP-HA-F	CTTGGTACCGAGCTCGGATCCATGCATCCT GGGCGAACTACT
pcDNA-M1AP-HA-R	TGCTGGATATCTGCAGAATTCTTAGGGCCT TGAGGGATCCT
pcDNA-REDIC1-HA-F	CTTGGTACCGAGCTCGGATCCATGAATTG GGTCGGGGGG
pcDNA-REDIC1-HA-R	TGCTGGATATCTGCAGAATTCTTAGTTGTT CTTATGGATTTCCTGCC
pCMV-mSHOC1-FLAG-F	CTTGCGGCCGGCCGCGAATTCGAATTCAT GGCATTGAACGGA
pCMV-mSHOC1-FLAG-R	CAGGGATGCCACCCGGGATCCTCTAGATA AAAACTTCAGCCGGG
pcDNA-mTEX11-HA-F	CTTGGTACCGAGCTCGGATCCATGGACCG CATTACTGACTTTTACT
pcDNA-mTEX11-HA-R	TGCTGGATATCTGCAGAATTCTTACAGATG GTTTTGAGCTGCCA
pcDNA-mTEX11-MYC-F	CTTGGTACCGAGCTCGGATCCATGGACCG CATTACTGACTTTTACT
pcDNA-mTEX11-MYC-R	TGCTGGATATCTGCAGAATTCTTACAGATG GTTTTGAGCTGCCA
pcDNA-mSPO16-HA-F	CTTGGTACCGAGCTCGGATCCATGGATGA GCGTAGAGGAAAAGA
pcDNA-mSPO16-HA -R	TGCTGGATATCTGCAGAATTCTCACTCTCC TGAGTCTGCACTAACTG
pcDNA-mM1AP-HA-F	CAGGGATGCCACCCGGGATCCATGAACCG AAGGAAAACTACTAGTAGAG
pcDNA-mM1AP-HA-R	CTTGCGGCCGGCCGCGAATTCTTAGGTGT GAGAAGGACGCTCC
pcDNA-mREDIC1-HA-F	CTTGGTACCGAGCTCGGATCCATGAACTG GGTCGGGGGC
pcDNA-mREDIC1-HA-R	TGCTGGATATCTGCAGAATTCTTAGAGTG AGTTACTTGTAGGTGTTTCCT
pCMV-SHOC1-XPF-FLAG-F	CTTGCGGCCGGCCGCGAATTCAAGTGATG CTACAAAAATGCTTTAACA
pCMV-SHOC1-XPF-FLAG-R	CAGGGATGCCACCCGGGATCCGGAACAG ATAGTGTCATTGAAATTCAA
pCMV-SHOC1-XPF-Mut-FLAG-F	CAGATAGCCGGTGCCAAGCATGTGCCAAG CATTTTGCCTCC
pCMV-SHOC1-XPF-Mut-FLAG-R	TTGGCACCGGCTATCTGACGCGGCTATCT GACGCTTGAATTTCA
pCMV-mSHOC1-XPF-FLAG-F	CTTGCGGCCGGCCGCGAATTCAAGTGATG CTGCAAAAATGCTTC
pCMV-mSHOC1-XPF-FLAG-R	CAGGGATGCCACCCGGGATCCCCAGAGG AGAGATGTGAAGAAAGG
pCMV-mSHOC1-XPF-Mut-FLAG-F	GTGCCAAGCATACTGTCTCCGTGCCAAGC ATACTGTCTCCTAGA
pCMV-mSHOC1-XPF-Mut-FLAG-R	AGACAGTATGCTTGGCACCGGGTGTCTGA AGCGGGGATCTGGGTGTCTGAAGCGGGG

	A
pCMV-SHOC1-FLAG-Venus-N-F	GTGAACCGTCAGAATTAACCATGGACTACAAAGACCATGACGG
pCMV-SHOC1-FLAG-Venus-N-R	CTCACCCCCCCGGACCCCCCAAAAAACCTCAGCCGAGTCTGC
pcDNA-TEX11-HA-Venus-C-F	TAGTCATCGCTATTACCATGGTGATGCGGTTTTGGCAGTAC
pcDNA-TEX11-HA-Venus-C-R	TTGTCCCCCCCGGACCCCCCATCTGACTTGCTCCAGTAGCCATG
pcDNA-TEX11-FLAG-Venus-N-F	AGGATGACGATGACAAGCTTATGGACAATGATGATTTTTTTCCA
pcDNA-TEX11-FLAG-Venus-N-R	CTCACCCCCCCGGACCCCCCCTAATCTGACTTGCTCCAGTAGCCA
pcDNA-C1orf146-HA-Venus-C-F	TAGTCATCGCTATTACCATGGTGATGCGGTTTTGGCAGTAC
pcDNA-C1orf146-HA-Venus-C-R	TTGTCCCCCCCGGACCCCCCATTTGGGTTAACTGAATCACTATTCAG
pCMV-mSHOC1-FLAG-Venus-N-F	AGGATGACGATGACAAGCTTATGGCATTGAACGGAAGAACTATG
pCMV-mSHOC1-FLAG-Venus-N-R	CTCACCCCCCCGGACCCCCCCTCATAAAAACTTCAGCCGGGTT
pcDNA-mTEX11-HA-Venus-C-F	TTCCAGATTACGCCAAGCTTATGGACCGCATTACTGACTTTTACT
pcDNA-mTEX11-HA-Venus-C-R	TTGTCCCCCCCGGACCCCCCCTTACAGATGGTTTTGAGCTGCCA
pcDNA-mTEX11-FLAG-Venus-N-F	AGGATGACGATGACAAGCTTATGGACCGCATTACTGACTTTTACT
pcDNA-mTEX11-FLAG-Venus-N-R	CTCACCCCCCCGGACCCCCCCTTACAGATGGTTTTGAGCTGCCA
pcDNA-SPO16-HA-Venus-C-F	TTCCAGATTACGCCAAGCTTATGGATGAGCGTAGAGGAAAAGA
pcDNA--SPO16-HA-Venus-C-R	TTGTCCCCCCCGGACCCCCCCTCACTCTCCTGAGTCTGCACTAACTG

Supplementary Table S2. Information of the antibodies.

Name of antibodies	Company	Catalog number	Host	Dilution
SYCP3	R&D	AF3750	Goat	IF (1:25) for human
SYCP3	Abcam	ab97672	Mouse	IF (1:200) for mice
SYCP3	Abcam	ab15093	Rabbit	IF (1:200) for mice
SYCP1	Gift from Liu Lab	/	Guinea pig	IF (1:200)
γH2AX	Millipore	2668445	Mouse	IF (1:500)
γH2AX	CST	9718	Rabbit	IF (1:500)
DMC1	Santa Cruz	sc-373862	Mouse	IF (1:200) for human
DMC1	Customed	/	Rabbit	IF (1:100) for mice
TRA98	Abcam	ab82527	Rat	IF (1:200)
pHH3	CST	9701	Rabbit	IF (1:200)
c-PARP	CST	9548	Mouse	IF (1:200)
MLH1	BD	51-1327GR	Mouse	IF (1:50)
H1T	Proteintech	18188-1-AP	Rabbit	IF (1:100)
HORMAD1	Proteintech	13917-1-AP	Rabbit	IF (1:200)
SPATA22	Proteintech	16989-1-AP	Rabbit	IF (1:100)
RPA2	CST	2208	Rat	IF (1:100)
RAD51	Customed	/	Rabbit	IF (1:100)
TEX11	Gift from Yu Lab	/	Goat	IF (1:50)
MSH4	Abcam	Ab58666	Rabbit	IF (1:50)
REDIC1	Gift from Shi Lab	/	Rabbit	IF (1:100)
M1AP	Gift from Shi Lab	/	Rabbit	IF (1:100)
HEI10	Gift from Liu Lab	/	Rabbit	IF (1:100)
POL II	Abcam	Ab5095	Rabbit	IF (1:100)
MDC1	Proteintech	24721-1-AP	Rabbit	IF (1:200)
BRCA1	Gift from Gai Lab	/	Rabbit	IF (1:100)
p-CHK1	CST	2348	Rabbit	IF (1:100)
BUB3	Abcam	ab133699	Rabbit	IF (1:100)
FLAG-tag	Sigma	F1804	Mouse	WB (1:5000)
HA-tag	CST	C29F4	Rabbit	WB (1:2000)
MYC-tag	CST	2278	Rabbit	WB (1:2000)
β-ACTIN	Proteintech	60008	Mouse	WB (1:5000)
Alexa Fluor® 488 Donkey	Invitrogen	A-21202	Donkey	IF (1:400)

anti-Mouse IgG (H+L)				
Alexa Fluor® 594 Donkey anti-Mouse IgG (H+L)	Invitrogen	A-21203	Donkey	IF (1: 400)
Alexa Fluor® 488 Donkey anti-Rabbit IgG (H+L)	Invitrogen	A-21206	Donkey	IF (1: 400)
Alexa Fluor® 594 Donkey anti-Rabbit IgG (H+L)	Invitrogen	A-21207	Donkey	IF (1: 400)
Alexa Fluor® 488 Donkey anti-Rat IgG (H+L)	Invitrogen	A-21208	Donkey	IF (1: 400)
Alexa Fluor® 488 Donkey anti-Goat IgG (H+L)	Invitrogen	A-11055	Donkey	IF (1: 400)

Supplementary Table S3. Clinical and semen characteristics in Chinese men with bi-allelic *SHOC1* variants

	Subject					
	P1	P2	P3	P4	P5	Reference
**Characteristics**						
Age (years)	29	30	25	34	33	/
Testicular Volume (Left) (ml)	10	15	10	10	15	12~15
Testicular Volume (Right) (ml)	10	20	10	10	20	12~15
FSH (IU/L)	12.44	3.9	4.83	3.32	5.2	1.27~19.26
LH (IU/L)	5.76	5.33	4.47	3.04	4.7	1.24~8.62
T (μg/L)	13.67	8.5	27.24	11.53	3.96	1.75~7.81
Karyotype	46,XY	46,XY	46,XY	46,XY	46,XY	46,XY
Y Chromosome Microdeletions	N	N	N	N	N	N
**Semen parameters**						
Semen volume (ml)						≥1.5
Sperm concentration (10^6/ml)	0	0	0	0	0	≥15
PR (%)	0	0	0	0	0	≥32
NP (%)	0	0	0	0	0	/
IM (%)	0	0	0	0	0	/
Centrifuged spermatozoa number (/ejaculate)	0	0	0	0	0	/

Abbreviations: FSH, follicle-stimulating hormone; LH, luteinizing hormone; T, testosterone; PR, progressive; NP, non-progressive; IM, immobility; N, normal phenotype.

Supplementary figure legends

Figure S1. Comparative interaction dynamics and assembly models of mouse and human SHOC1 complexes.

(A) Co-IP demonstrated the interaction of mouse TEX11 (detected via N-terminal MYC tag) with SPO16 (detected via N-terminal HA tag) in the presence or absence of SHOC1. Input lysates were included.

(B) Co-IP demonstrated the no interaction of human TEX11 (detected via N-terminal MYC tag) with C1orf146 (detected via N-terminal HA tag) in the presence or absence of SHOC1. Input lysates were included.

(C) Bi-FC revealed Venus fluorescence signals in all mouse SHOC1/TEX11, SHOC1/SPO16, TEX11/SPO16-coexpressing cells. Scale bars, 50 μm.

Figure S2. Specific regions of human and mouse SHOC1 required for interaction with binding partners.

(A,B) Schematic diagrams of truncated human (A) and mouse (B) SHOC1 protein constructs.

(C) Co-IP assays showing interactions between full-length human TEX11, M1AP, or REDIC1 (detected using N-terminal HA- or MYC-tags) and truncated human SHOC1 constructs (detected using an N-terminal FLAG-tag) in HEK293T cells. Input lysates were included.

(D) Co-IP assays demonstrating interactions between full-length mouse TEX11, M1AP, or REDIC1 (detected using N-terminal HA-tags) and truncated mouse SHOC1 constructs (detected using an N-terminal FLAG-tag) in HEK293T cells. Input lysates were included.

Figure S3. Effects of *SHOC1* variants assessed by *in vitro* analyses.

(A) WB analysis of 3×FLAG-SHOC1 fusion protein expression in HEK293T cells transfected with WT or mutant (M1 to M6) plasmids. Target protein bands are indicated by blue rectangles.

(B) Relative intensities quantified for WT and M1 mutant 3×FLAG-SHOC1 fusion protein expression in HEK293T cells using two-tailed Student's t-test; ns, not significant; error bars, mean ± SEM.

(C) Reverse transcription-PCR products of pcMINI vectors expressing WT or c. G2738-1A *SHOC1* minigenes (containing genomic sequences: intron [179 bp]-exon 21 [118 bp]-intron [112 bp]) in HEK293T cells.

(D) Sanger sequencing analysis of splicing products. WT transcripts exhibited canonical splicing, while mutant transcripts underwent aberrant splicing, resulting in exon 21 skipping.

(E) Schematic representation of canonical (WT) and aberrant (mutant) splicing, and predicted protein truncation caused by the c.G2738-1A variant in *SHOC1*. The red asterisk indicates the variant site.

Figure S4. Meiotic arrest phenotype in the patient carrying the missense variant (p.Q590R) within the XPF-like domain in *SHOC1*.

(A) H&E staining of testicular sections from the proband (left) and an OA control (right). Scale bars, 50 μm.

(B) IF staining of testicular sections from the proband and an OA control with DMC1 (green) and PNA (red). Scale bars, 50 μm.

(C) IF staining of testicular sections from the proband and an OA control with SYCP3 (green) and γH2AX (red). Scale bars, 50 μm.

(D) Representative images of spread spermatocytes from the proband and an OA control co-stained with SYCP3 (green) and γH2AX (red). Scale bars, 20 μm.

(E) Schematic diagram of meiotic arrest and male infertility caused by bi-allelic *SHOC1* variants.

Figure S5. *Shoc1* KI caused follicular dysplasia and female infertility.

(A) Representative image comparing ovary size in adult *Shoc1* KI homozygous (*Shoc1*$^{KI/KI}$) mice and littermate controls (*Shoc1*$^{KI/+}$).

(B) Ovary-to-body weight ratios quantified for *Shoc1* KI homozygous (*Shoc1*$^{KI/KI}$) mice and littermate controls (*Shoc1*$^{KI/+}$) using two-tailed Student's t-test; **** $P < 0.0001$; error bars, mean ± SEM.

(C) H&E staining of ovary sections from adult *Shoc1* KI homozygous (*Shoc1*$^{KI/KI}$) mice and littermate controls (*Shoc1*$^{KI/+}$). Scale bars, 100 μm.

Figure S6. Meiotic prophase I analysis.

(A) Representative images of spread spermatocytes from adult *Shoc1* KI homozygous (*Shoc1*$^{KI/KI}$) mice and littermate controls (*Shoc1*$^{KI/+}$) co-stained with SYCP3 (green) and γH2AX (red). Scale bars, 10 μm.

(B) Proportions of spermatocytes at defined substages using two-tailed Student's t-test; *** $P < 0.001$; * $P < 0.05$; ns, not significant; error bars, mean ± SEM; n, the total number of nuclei analyzed.

Figure S7. *Shoc1* KI leads to MMI arrest through pachytene checkpoint bypass and SAC activation.

(A-C) Representative images of spread spermatocytes from adult *Shoc1* KI homozygous (*Shoc1*$^{KI/KI}$) mice and littermate controls *(Shoc1*$^{KI/+}$) co-stained with SYCP3 (Red) and BRCA1 (green, A), SYCP3 (Red) and p-CHK1 (green, B), BUB3 (green) and DAPI (blue, C). Scale bars, 10 μm.

(D) Quantification of BUB3 positive signals in pachytene spermatocytes from adult *Shoc1* KI homozygous (*Shoc1*$^{KI/KI}$) mice and littermate controls (*Shoc1*$^{KI/+}$) using two-tailed Student's t-test; ** $P < 0.01$; error bars, mean ± SEM; n, the total number of nuclei analyzed.

Figure S8. DSBs repair defects in *Shoc1* KI spermatocytes.

(A,C,E,G) Representative images of spread spermatocytes from adult *Shoc1* KI homozygous (*Shoc1*$^{KI/KI}$) mice and littermate controls (*Shoc1*$^{KI/+}$) co-stained with SYCP3 (green) and DMC1 (red, A), RAD51 (red, C), RPA2 (red, E), SPATA22 (red, G). Scale bars, 10 μm.

(B,D,F,H) Quantification of DMC1 (B), RAD51 (D), RPA2 (F), SPATA22 (H) foci per cell at indicated meiotic stages using two-tailed Student's t-test; **** $P < 0.0001$; ns, not significant; error bars, mean ± SEM; n, the total number of nuclei analyzed.

Figure S9. Visualization of both human and mouse SHOC1 mutants through molecular docking.

(A,B) Structural model of WT (A) and mutant (B) human SHOC1 (predicted by AlphaFold3)/D-loop (PDB ID: 7JY7) complexes generated using the HDOCK server. Residue Q590 and its mutation R590 are highlighted in red dash rectangles, with hydrogen bond interactions indicated by blue dash lines. Confidence scores above 0.7 indicate high probability of binding.

(C,D) Zoom the view of Q590 (C) and its mutation R590 (D) residues.

(E) Comparison of docking scores between WT and mutant human SHOC1/D-loop complexes using two-tailed Student's t-test, with lower (more negative) docking scores corresponding to increased binding energy; **** $P < 0.0001$; ns, error bars, mean ± SEM.

(F,G) Structural model of WT (F) and mutant (G) mouse SHOC1 (predicted by AlphaFold3)/D-loop (PDB ID: 7JY7) complexes generated using the HDOCK server. Residue Q646 and its mutation R646 are highlighted in red dash rectangles, with hydrogen bond interactions indicated by blue dash lines. Confidence scores above 0.7 indicate high probability of binding.

(H,I) Zoom the view of Q646 (H) and its mutation R646 (I) residues.

(J) Comparison of docking scores between WT and mutant mouse SHOC1/D-loop complexes using two-tailed Student's t-test, with lower (more negative) docking scores corresponding to increased binding energy; **** $P < 0.0001$; ns, error bars, mean ± SEM.

Figure S10. Isolation of pachytene spermatocytes by modified STA-PUT.

(**A**) Schematic workflow of the STA-PUT density gradient protocol for isolating pachytene spermatocytes from adult *Shoc1* KI homozygous (*Shoc1*$^{KI/KI}$) mice.

(B) Representative images of enriched cell fractions co-stained with SYCP3 (red) and γH2AX. Scale bars, 10 μm.

(**C)** Quantification of germ cell type distributions across fractions from adult *Shoc1*$^{KI/KI}$ male mice; mean ± SEM.

Figure S11. *Shoc1* KI induced excessive accumulation of DNA damage response (DDR) factors and establishment of MSUC on autosomes.

(A,C) Representative images of spread spermatocytes from adult *Shoc1* KI homozygous (*Shoc1*$^{KI/KI}$) mice and littermate controls (*Shoc1*$^{KI/+}$) co-stained with SYCP3 (green) and POL II (red, A), MDC1 (red, C). Scale bars, 10 μm.

(B,D) Quantification of POL II (B), MDC1 (D) foci per cell at indicated meiotic stages using two-tailed Student's t-test; **** $P < 0.0001$; ns, not significant; error bars, mean ± SEM; n, the total number of nuclei analyzed.

Figure S12. The pseudotime analysis showed similar developmental trajectory of spermatocytes in *Shoc1* KI and control groups.

The pseudotime trajectory plot of spermatocytes from adult *Shoc1* KI homozygous (*Shoc1*$^{KI/KI}$) mice and littermate controls (*Shoc1*$^{KI/+}$).
